# Rapid Patient-Side Evaluation of Endothelial Glycocalyx Thickness in Healthy Sedated Cats Using GlycoCheck® Software

**DOI:** 10.3389/fvets.2021.727063

**Published:** 2022-01-03

**Authors:** Ivayla D. Yozova, Leonel A. Londoño, Kristina K. Millar, Hiroki Sano, Karin Weidgraaf, Neroli A. Thomson, John S. Munday

**Affiliations:** ^1^School of Veterinary Science, Massey University, Palmerston North, New Zealand; ^2^Department of Small Animal Clinical Sciences, College of Veterinary Medicine, University of Florida, Gainesville, FL, United States; ^3^Veterinary Teaching Hospital, College of Veterinary Medicine, University of Georgia, Athens, GA, United States; ^4^Centre for Feline Nutrition, Massey University, Palmerston North, New Zealand

**Keywords:** microcirculation, capillaries, perfused boundary region, sidestream dark field videomicroscopy, feline

## Abstract

The endothelial glycocalyx (EG) determines transvascular fluid fluxes, and influences inflammation, coagulation, and capillary blood flow. The GlycoCheck® software calculates EG thickness using sidestream dark field videomicroscopy recordings. This method has not been evaluated for use in cats. The aim of the present study was to evaluate the use of GlycoCheck® for estimating EG thickness in healthy cats, and to investigate the variability of EG thickness in this population. One hundred and one healthy research-purposed cats were included in the study. The cats were sedated, and a handheld videomicroscope, connected to GlycoCheck® software, was used to evaluate the sublingual microvasculature. The parameters measured included perfused boundary region (PBR, an indirect measurement of EG thickness) in vessels between 5 and 25 μm in diameter, valid vessel density, percentage red blood cell filling, and median red blood cell column width. Heart rate, respiratory rate, pulse oximetry and oscillometric blood pressure readings were also recorded. There were 35 neutered male cats, 11 intact males, 38 neutered females, and 17 intact females. The average age was 63 months (range, 11–160 months). Tolerance intervals for PBR (vessel diameter 5–25 μm) were 1.89–3.00 μm (95% CI, lower limit 1.76–2.04, upper limit 2.83–3.13 μm); for valid vessel density were 73.33–333.33 μm/mm^2^ (95% CI, lower limit 77.00–99.33, upper limit 312.67–350.33 μm/mm^2^); for percentage red blood cell filling were 59.85–85.07% (95% CI, lower limit 58.97–63.33, upper limit 83.07–88.20 %); and for median red blood cell column width were 5.63–8.59 μm (95% CI, lower limit 5.28–6.07, upper limit 8.14–9.51 μm). There was a negative association between median red blood cell column width and body weight (*p* = *0.007*). The median red blood cell column was significantly wider in intact females when compared to spayed females (*p* = *0.033*). The GlycoCheck® analysis was easily performed in healthy sedated cats. Clinical variables did not have an effect on the EG thickness. These results suggest that this technique could be valuable for evaluation of the EG and microvascular parameters in cats.

## Introduction

The endothelial glycocalyx (EG) lines the endothelial surface of mammalian blood vessels, and consists of complex sugars bound directly to the endothelial membrane, and to each other. Furthermore, it incorporates a variety of smaller molecules (e.g. albumin, antithrombin) relevant to maintenance of blood vessel structure and function (1). The EG is a major determinant of transvascular fluid fluxes, it participates in local regulation of capillary blood flow, and influences inflammation and coagulation (2, 3). Systemic inflammatory states such as sepsis, trauma, ischemia-reperfusion injury, hypervolemia, and chronic illnesses can damage the EG. This can allow more fluid to leak from compromised blood vessels, exacerbating interstitial oedema. Damage to the EG can also alter capillary blood flow, promote inflammation, and trigger intravascular coagulation (4–7). The significance of EG integrity and functionality in disease has been increasingly recognised in recent years, and altered EG has been associated with increased morbidity and mortality risk in critical illness in people (7–11).

**Figure 1:**
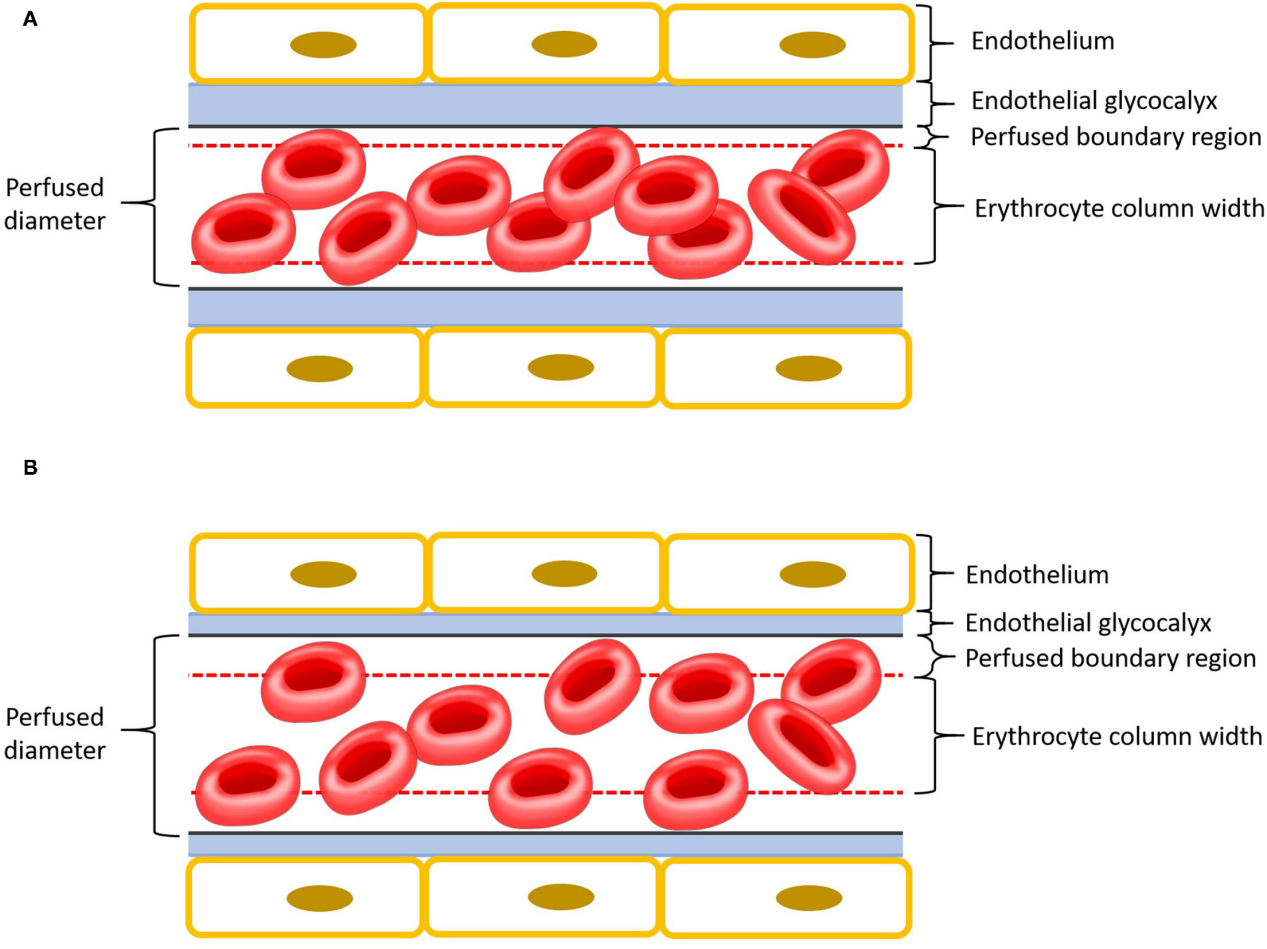
Schematic representation of a capillary with endothelium is in yellow, endothelial glycocalyx in blue, perfused boundary region in red dashed lines, and erythrocytes in red. Please note that the different components of this cartoon are scaled for emphasis. **(A)** Intact endothelial glycocalyx characterized by a small perfused boundary region. **(B)** Damaged (thinner) endothelial glycocalyx characterized by a large perfused boundary region.

One method for assessment of EG integrity includes measurement of the plasma concentration of shed EG constituents (12). Membrane-bound glycoproteins such as syndecan-1 are cleaved from the endothelial surface when the EG is damaged. The resultant increase of these glycoproteins can be measured in the plasma (6, 10, 12). Elevated plasma EG glycoprotein concentrations have been associated with increased morbidity and mortality in people with conditions leading to systemic inflammation (13, 14). However, while some evidence suggests syndecan-1 may be a useful prognostic indicator in people (9, 12), a relationship between syndecan-1 concentration and disease severity has not been seen in all studies (15). Alternatively, EG thickness can be estimated using sidestream dark field videomicroscopy (SDFV) and proprietary software (GlycoCheck®) (16). The software allows EG thickness to be determined by analysing multiple SDFV images and calculating the parameter perfused boundary region (PBR). Larger PBR represents thinner EG and vice versa (Figure 1). This tool has recently been validated for use in dogs (17). Although SDFV has been previously used to assess microcirculaton in cats, dogs and people, using GlycoCheck® software to estimate EG thickness has not been done in felines (18–22).

The aim of the present study was to investigate whether EG thickness of cats can be evaluated using SDFV and the GlycoCheck® software. A secondary aim was to determine the tolerance intervals for GlycoCheck® parameters. The hypothesis was that estimation of EG thickness using the GlycoCheck® software would be feasible in cats.

## Materials and Methods

### Animals

This project was approved by Massey University's Animal Ethics Committee (MUAEC Protocol 18/126). One hundred and eight cats from the Centre for Feline Nutrition, Massey University, New Zealand were enrolled in the study. They were considered to be healthy based on no history of ongoing disease, physical examination findings, and blood work (complete blood count and serum biochemistry, including T4) results. Cats that showed signs of illness were excluded from the study. Feasibility had been investigated on a small number of cats (*n* = 3) prior to initiation of the project (see Supplementary Material).

### Sedation and Monitoring

All animals were sedated with butorphanol 0.3 mg/kg SC for intravenous catheter placement in the left or right cephalic vein. Twelve cats were additionally sedated with medetomidine 10 μg/kg SC, based on previous handling challenges. Propofol was then administered intravenously to achieve heavy sedation (relaxation of jaw tone) to effect. The sedation was maintained using a continuous rate infusion of propofol (0.05–0.2 mg/kg/min) for the duration of the procedure. Sedation was overseen by a board-certified anaesthesiologist and clinical variables such as heart rate (HR), respiratory rate (RR), mucous membrane colour, capillary refill time, temperature, pulse oximetry (SpO_2_), and oscillometric blood pressure (oBP) readings were monitored.

### Image Acquisition

**Figure 2:**
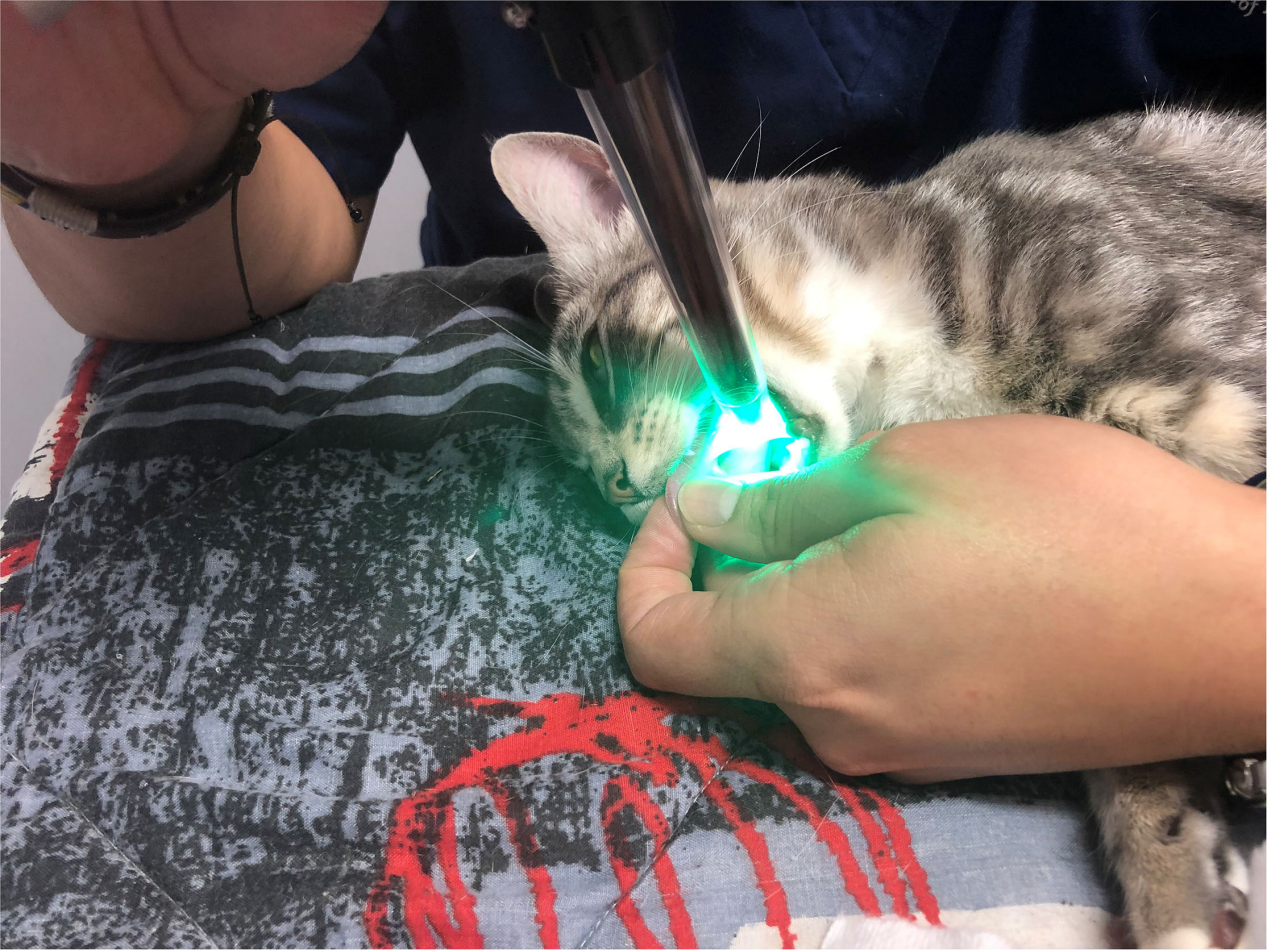
Probe positioning of the sidestream dark field videomicroscopy camera for GlycoCheck® analysis.

**Figure 3:**
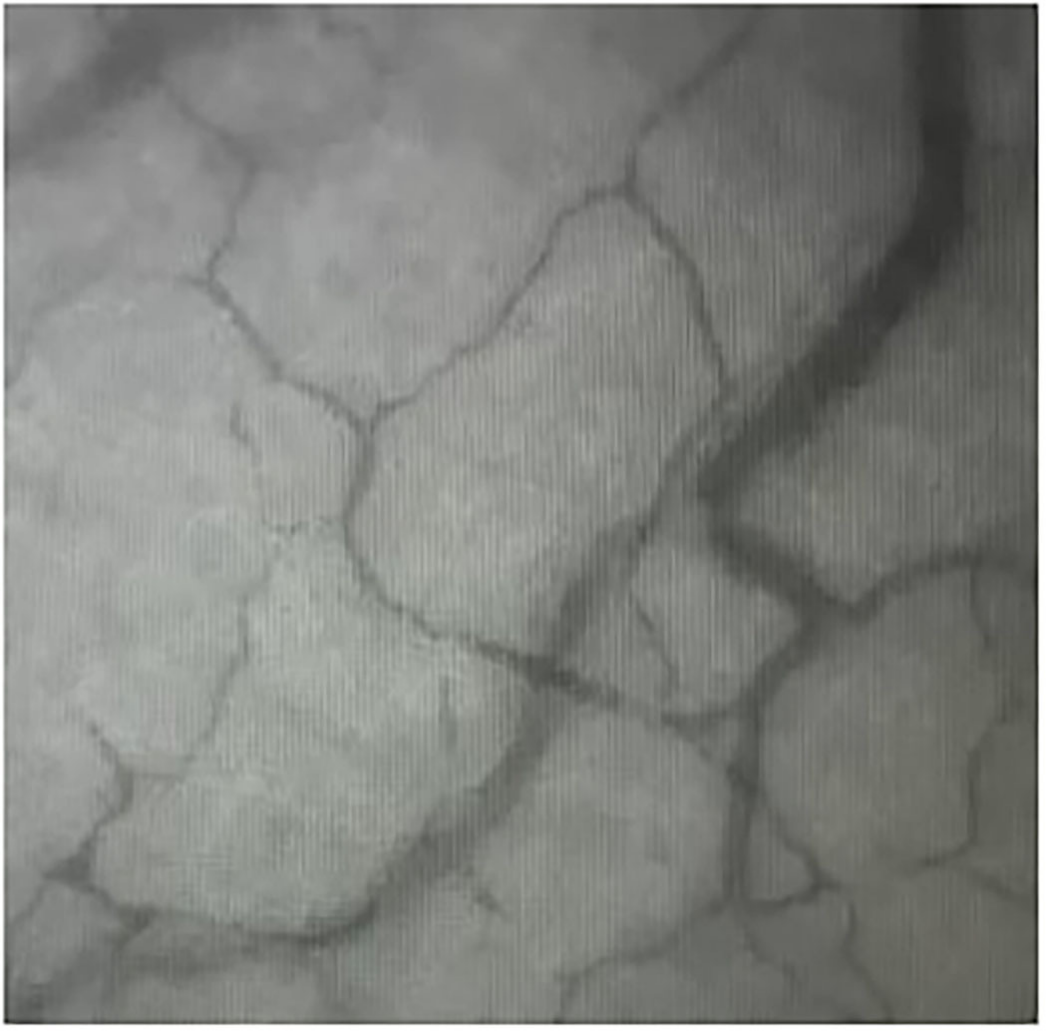
Sidestream dark field videomicroscopy image of the sublingual mucosal capillaries in a cat.

Cats were placed in lateral recumbency, and the tongue was positioned such that the sublingual mucosa was exposed at the base of the tongue (Figure 2). A hand-held SDFV camera connected to a laptop with GlycoCheck® software (MicroVascular Health Solutions, USA) was placed in contact with the sublingual mucosa by the operator (LL). Three video recordings were taken from different sublingual mucosal sites (Figure 3). The software automatically determines whether a sufficient number of suitable images have been acquired prior to moving to an additional site. This is signalled through a progress bar on the monitor display. Additionally, the software corrects for operator-induced errors such as movement and focus artefacts.

### GlycoCheck® Analysis

The method for measuring and calculating GlycoCheck® parameters has been described elsewhere (23). Briefly, the SDFV camera emits green light at 540 nm wavelength which is absorbed by haemoglobin in circulating red blood cells (RBC). Therefore, vessels appear dark on a bright background. The software automatically identifies vessels that are <30 μm in diameter by analysing the contrast between the RBC and the background. The software then subdivides vessels into 10 μm major vascular segments and subsequently records 40 frames containing on average 300 segments. The software ensures measurements are performed on vascular segments of good quality. Firstly, the vascular segments from the first frame of each recording are further divided by 0.5 μm markers into minor segments and each segment is analysed for sufficient contrast. Major segments with > 60% of minor segments with sufficient contrast are considered valid vascular segments. Valid vessel density per site is then expressed by multiplying 10 μm by the number of valid vascular segments identified. Secondly, RBC column widths are measured for all 40 frames of each recording including minimal column width, column position, and signal-to-noise ratio. The software then calculates the percentage of major vascular segments with RBC present during all 40 frames recorded to determine the percentage of RBC filling. After those quality checks, the dynamic lateral position of RBC per column width is determined. A cumulative distribution is calculated using the intensity profiles of the distribution of RBC column widths. A median column width is then calculated for this distribution. Linear regression analysis on the median column width is performed to derive the perfused diameter. Finally, the PBR, an estimate of EG thickness, is calculated as the distance between the median RBC column width and the perfused diameter ([Perfused diameter—median RBC column width]/2). Reported GlycoCheck® parameters, therefore, include valid vessel density (VVD), percentage of RBC filling (% RBC filling), median RBC column width (Median P50), perfused vessel diameter (D_perf_) and PBR for vessels with diameter from 5 to 25 μm. The PBR is further established for vessels with diameters from 5 to 9, 10 to 19, and 20 to 25 μm. All GlycoCheck® parameters are described in Table 1.

**Table 1:** Description and interpretation of GlycoCheck® parameters.

**Parameter**	**Description**	**Interpretation**
Valid Vessel Segment^*^ (VVS)	Determined by the amount of minor vascular segments (0.5 μm) within a major vascular segment (10 μm) with sufficient background contrast	Identifies adequately perfused vessel segments
Valid Vessel Density (VVD)	Determined by multiplying the number of valid vessel segments by 10 μm	Identifies adequate microvascular perfusion in the examined regions
Percentage of RBC filling (% RBC filling)	Percentage of vascular segments with RBC in them in all 40 frames per recording	Identifies adequate microvascular perfusion in the examined regions
Median RBC column width (P50)	Calculated using the intensity profiles of distribution of RBC column widths in segments based on the dynamic lateral position of RBC in the column	Represents the portion of RBC column without RBC lateralization. Used to calculate endothelial glycocalyx thickness
Perfused vessel diameter (D_perf_)	Derived from the median RBC column width using linear regression	Represents the outer limit of lateral movement of RBC. Used to calculate endothelial glycocalyx thickness
Perfused boundary region (PBR)	Calculated using the formula (Perfused diameter—median RBC column width)/2	Estimation of endothelial glycocalyx thickness. Reported for vessels with diameters of 5–9 μm, 10–19 μm, and 20–25 μm separately, and as an average diameter of 5–25 μm

* *Not automatically reported*.

### Statistical Analyses

Statistical analyses were performed by an independent statistical consultant using the commercially available software program JMP (Version 16, SAS Institute, Cary, NC, USA). All variables were analysed for normality using the Kolmogorov- Smirnov method and reported as mean ± standard deviation. Covariate analysis for continuous and non-continuous variables using a simple linear regression and one way ANOVA were performed, respectively, to assess relationships between microvascular measurements, and independent clinical variables monitored during sedation (HR, RR, SpO_2_, oBP), or macrovascular monitored parameters. If the ANOVA was significant, then Tukey's multiple comparison procedure was used to determine differences in levels for categorical variables. Restricted maximum likelihood estimation was used for intra-individual and inter-individual variance component estimates. Non-parametric tolerance estimates and point estimates confidence intervals were estimated using 2,500 bootstrap samples from the average of three measurements. For all analyses a *P* < 0.05 was considered significant. A *post hoc* sample size calculation was performed for clinical variables monitored during sedation with a power set to 80% and α = 0.05.

## Results

Complete data acquisition was possible from 101 cats. Seven cats were excluded from the study due to recording malfunction (*n* = 3), development of post-sedation heart murmur (*n* = 1), lethargy (*n* = 1) and elevated T4 (*n* = 2). Of those included, 46 were male (11 intact and 35 neutered) and 55 were female (38 intact and 17 spayed). Mean age ± standard deviation (SD) was 67 ± 46 months. Mean body weight ± SD was 3.58 ± 0.79 kg.

Average image acquisition time per site was 167 seconds (range, 45–269 s) and per cat was 490 s (range, 118–808 s). The average number of valid recordings obtained per subject were 50 (range, 25–64). Success rate for image acquisition and analysis was 93.5%.

Heart rate, RR, SpO_2_, oBP of all the cats remained within normal limits throughout the short period of sedation required for image acquisition (see Supplementary Material).

Mean ± SD values for GlycoCheck® parameters are included in Table 2. There was no significant association between clinical variables monitored during sedation (HR, RR, SpO_2_, oBP) and VVD, % RBC filling, median P50, Dperf, and PBR. Furthermore, there was no significant association between GlycoCheck® parameters of the cats that received medetomidine (*n* = 12) compared to the GlycoCheck® parameters of those that did not receive medetomidine (*n* = 89). There was no significant association between age and GlycoCheck® parameters. However, there was a negative association between median P50 and body weight (R^2^ = 0.029; *p* = *0.007*). Additionally, median P50 was significantly wider (R^2^ = 0.087; *p* = *0.033*) in intact females compared to spayed females. Tolerance intervals for GlycoCheck® parameters are presented in Table 3. Variance component estimates are presented in Table 4. A comparison of GlycoCheck® parameters in healthy cats, dogs, and people is presented in Table 5. All statistical analyses are presented in the Supplementary Material.

**Table 2:** GlycoCheck® parameters in a population of healthy cats (*n* = 101).

**Parameter**	**Mean ± SD**	**SE**	**Min**	**Max**
VVD (μm/mm^2^)	173.00 ± 64.80	6.50	77.00	350.00
% RBC Filling	72.70 ± 6.16	0.61	60.00	88.20
PBR 5–25 (μm)	2.39 ± 0.26	0.03	1.76	3.13
PBR 5–9 (μm)	1.34 ± 0.15	0.02	0.91	1.94
PBR 10–19 (μm)	2.70 ± 0.30	0.03	2.05	3.51
PBR 20–25 (μm)	2.92 ± 0.57	0.06	1.51	5.22
Median P50 (μm)	7.00 ± 0.68	0.07	5.28	9.51
D_perf_, 2PBR + P50 (μm)	11.80 ± 0.88	0.09	9.51	15.20

*D_perf_, Perfused Vessel Diameter; Median P50, Median Red Blood Cell Column Width; PRB, Perfused boundary region; % RBC Filling, Percentage Red Blood Cell Filling; VVD, Valid Vessel Density; SD, Standard Deviation; SE, Standard Error*.

**Table 3:** Tolerance intervals for GlycoCheck® parameters in a population of healthy cats (*n* = 101).

**Parameter**	**Lower limit**	**95% CI**	**Upper limit**	**95% CI**
VVD (μm/mm^2^)	73.33	77.00–99.33	333.33	312.67–350.33
% RBC Filling	59.85	58.97–63.33	85.07	83.07–88.20
PBR 5–25 (μm)	1.89	1.76–2.04	3.00	2.83–3.13
PBR 5–9 (μm)	0.97	0.91–1.08	1.58	1.55–1.64
PBR 10–19 (μm)	2.11	2.05–2.25	3.48	3.25–3.51
PBR 20–25 (μm)	1.87	1.51–2.26	4.02	3.94–5.23
Median P50 (μm)	5.63	5.28–6.07	8.59	8.14–9.51

*CI, Confidence Intervals; Median P50, Median Red Blood Cell Column Width; PBR, Perfused boundary region; % RBC Filling, Percentage Red Blood Cell Filling; VVD, Valid Vessel Density*.

**Table 4:** Variance component estimates for GlycoCheck® parameters in a population of healthy cats (*n* = 101).

**Parameter**	**Inter-individual variance components**				**Intra-individual variance components**			
	**Component**	**SE**	**95% CI**	**% of Total Variance**	**Component**	**SE**	**95% CI**	**% of Total Variance**
VVD	3170.18	611.86	1970.96–4369.40	50.13	3153.78	322.75	2605.76–3895.88	49.87
% RBC Filling	21.82	5.59	10.87–32.77	32.22	45.91	4.70	37.93–56.73	67.78
PBR 5–25	0.03	0.01	0.01–0.05	26.48	0.09	0.01	0.08–0.12	73.52
PBR 5–9	0.01	<0.01	0.01–0.02	29.52	0.03	<0.01	0.02–0.04	70.48
PBR 10–19	0.05	0.01	0.02–0.08	28.43	0.12	<0.01	0.10–0.15	71.57
PBR 20–25	0.05	0.05	−0.05–0.15	6.98	0.69	0.07	0.56–0.85	93.02
Median P50	0.29	0.07	0.16–0.43	35.41	0.53	0.05	0.44–0.66	64.59

*CI, Confidence Intervals; Median P50, Median Red Blood Cell Column Width; PBR, Perfused boundary region; % RBC Filling, Percentage Red Blood Cell Filling; VVD, Valid Vessel Density; SE, Standard Error*.

**Table 5:** Comparison of GlycoCheck® parameters in cats, dogs and people expressed as mean ± standard deviation.

**Parameter**	**Cats (*n* = 101)**	**Dogs (*n* = 54) (17)**	**People (*n* = 915) (23)**
VVD (μm/mm^2^)	173.00 ± 64.80	2030.68 ± 733.41	3213.00 ± 691.00
% RBC Filling	72.70 ± 6.16	77.4 ± 6.22	73.20 ± 5.00
PBR 5–25 (μm)	2.39 ± 0.26	2.04 ± 0.31	2.14 ± 0.25
Median P50 (μm)	7.00 ± 0.68	7.90 ± 0.85	10.56 ± 1.12
D_perf_ (μm)	11.80 ± 0.88	11.97 ± 1.05	14.84 ± 1.18

*D_perf_, Perfused Vessel Diameter; Median P50, Median Red Blood Cell Column Width; PBR, Perfused boundary region; % RBC Filling, Percentage Red Blood Cell Filling; VVD, Valid Vessel Density*.

## Discussion

This study demonstrated that measuring microcirculatory parameters such as VVD, % RBC filling, median P50, and D_perf_ using SDFV and the GlycoCheck® software is feasible and easily performed in healthy sedated cats. Furthermore, PBR can be calculated using these measured parameters in an acceptable period of time under heavy sedation with a high success rate. Effects of body weight and neuter status on median RBC column width were found. However, those did not alter the PBR and therefore the estimated EG thickness.

The importance of microcirculatory derangements, including EG damage in the context of the pathophysiology of circulatory shock has been brought forward in recent years (24, 25). Domestic cats present a challenge for circulatory assessment with tools designed for people and dogs, due to their small size. Furthermore, they typically present in a hypodynamic shock state, manifested as bradycardia, hypothermia, and hypotension, making it difficult to rely on conventional monitoring tools (26). There is increasing evidence that microcirculatory abnormalities can be present beyond macrohemodynamic stabilisation. Therefore, a tool that robustly evaluates the microcirculation and EG thickness specifically in a species where macrocirculatory evaluation is already challenging might prove particularly useful. GlycoCheck® parameters were evaluated in a large population of cats—not only to account for potential age and gender differences, but to also establish tolerance intervals. Tolerance intervals are a crucial step in generating expected values of a given parameter in healthy individuals. As such, they allow for identifying abnormalities in sick patients in the clinical setting and facilitate comparison and reproducibility in further research.

In this study, all cats were heavily sedated for GlycoCheck® evaluation because they were considered unlikely to tolerate having the SDFV camera in the oral cavity. Twelve cats required additional sedation using medetomidine as they were difficult to handle despite using Low Stress Handling®. However, the addition of this drug did not significantly alter any of the GlycoCheck® parameters. These results suggest that the method of sedation may not be a confounding factor for using GlycoCheck® in clinical practice, although additional studies using a greater range of sedation protocols are required to confirm this. Our results are consistent with a previous study in healthy dogs where the different sedation protocols also did not seem to alter GlycoCheck® parameters (17). Additionally, the need to sedate or anaesthetize a cat for image acquisition might preclude the use of the device in debilitated critically ill patients, because of the anaesthetic risk. However, evaluation of EG integrity would be most valuable in such patients. Therefore, evaluation of alternative image acquisition sites may prove helpful. Overall, changes in HR, RR, SpO_2_, oBP were not associated with significant changes in the GlycoCheck® parameters measured during this investigation. The cats were sedated for a brief period of time, and the clinical variables monitored during sedation remained close to normal. This might explain the lack of effects of those on GlycoCheck® parameters. Noteworthily, only a *post hoc* sample size calculation was made. It determined that the study was underpowered for some clinical variables monitored during sedation (see Supplementary Material). However, the sample size was sufficient to detect differences in mean arterial pressure, which is a measured parameter potentially affecting the microcirculation.

In people, PBR has been reported to increase with age (27). Such an association with PBR and age was not observed in this population of cats, despite the cats having an age range of 11–160 months. Whether this is a species-specific reflection of good vascular health, or rather the absence of age-related vascular comorbidities often seen in people is unknown. Furthermore, the study might have been underpowered to detect such differences.

Women have been found to have thinner EGs than men and their EG structure varies somewhat with their menstrual cycle (23, 28, 29). Typically, pet cats are neutered or spayed, therefore hormone-related gender dimorphism might not be a significant factor for their EG integrity. Nonetheless, the lack of sex hormones might result in changes in EG structure and functionality. While no significant effects were found for the estimated EG thickness in females compared to male cats, P50 was significantly wider in intact compared to spayed females. This signifies less RBC lateralization in the intact females. Increased RBC lateralisation could be interpreted as an early marker of EG damage according to a recent study in people (23).

In previous studies in people, an increase in body mass index was associated with evidence of potential capillary damage, manifested as increased perfused-to-total capillary density (27). In the present study, P50 decreased as body weight increased. It should be noted that although body mass and gender status were associated with changes in P50, these were not associated with any detectible differences in the estimated EG thickness. Whether the neuter status and body weight differences in domestic cats are related to a milder form of EG damage not affecting its actual thickness is currently uncertain.

Although direct comparison between different studies is not possible, the results of the present study suggest that vessel density is higher in the oral submucosa in dogs and people compared to cats (17, 23). Anatomical differences in capillary density between species have been previously reported for several vascular beds (30, 31). Furthermore, previous studies measuring perfused vessel density have also reported differences between species (19, 21, 22, 32). This signifies that caution is warranted when extrapolating results from one species to another, including experimental to clinical data.

Sidestream dark field videomicroscopy uses polarised light emitted in the absorption wavelength of haemoglobin giving RBC their dark appearance on a contrasting light background. Therefore, peculiarities of feline haemoglobin, such as three pH-dependent forms of haemoglobin and eight sulfhydryl groups, might have affected the results (33). Furthermore, feline RBC contain haemoglobin that has a lower oxygen affinity compared to human and canine haemoglobin. However, while the oxygen affinity is lower, the oxygen content in RBC appears to be similar between cats and people (34). Additionally, absorbance spectra seem to be similar between cats, dogs, horses, pigs, and people; suggesting that peculiarities of feline haemoglobin would not affect measurements (35).

In the present study, sublingual microcirculation was evaluated in healthy sedated cats. While perfusion of this area is expected to be adequate in such subjects, this might not be the case in patients with circulatory impairment. Indeed, using the sublingual microcirculation as a surrogate for all capillaries represents a major limitation of the technique as damage to the EG might not be equally represented during disease processes. Furthermore, hypoxia or severe anaemia might also affect results in sick patients (36). Image acquisition was performed by a single operator in this study, which represents another major limitation. In a previous study in people, intra and inter-rater reproducibility was found to be poor (8). The GlycoCheck® is a novel software estimating EG thickness. A comparison with a gold standard would have increased the validity of the obtained measurements. However, currently there is no gold standard methodology to evaluate EG dimensions, integrity, or functionality.

## Conclusion

Based on this study, estimating EG thickness in healthy sedated cats was feasible using SDFV and the GlycoCheck® software. This is the first study evaluating the EG in the feline species and will serve as a stepping stone for investigating the feline EG in health and disease. Tolerance intervals were established as a critical step for future studies in domestic cats.

## Data Availability Statement

The raw data supporting the conclusions of this article will be made available by the authors, without undue reservation.

## Ethics Statement

The animal study was reviewed and approved by Massey University Animal Ethics Committee (MUAEC Protocol 18/126).

## Author Contributions

IY, LL, HS, and KW contributed to conception and design of the study. IY wrote the first draft of the manuscript. IY, LL, KM, and HS wrote sections of the manuscript. NT and JM revised the manuscript. All authors contributed to manuscript revision, read, and approved the submitted version.

## Funding

This project was funded by the Massey University Research Fund - RM 21500.

## Conflict of Interest

The authors declare that the research was conducted in the absence of any commercial or financial relationships that could be construed as a potential conflict of interest.

## Publisher's Note

All claims expressed in this article are solely those of the authors and do not necessarily represent those of their affiliated organizations, or those of the publisher, the editors and the reviewers. Any product that may be evaluated in this article, or claim that may be made by its manufacturer, is not guaranteed or endorsed by the publisher.
