# Supplementary material for: Rapid Patient-Side Evaluation of Endothelial Glycocalyx Thickness in Healthy Sedated Cats Using GlycoCheck® Software
Source: Front Vet Sci. 2022 Jan 3;8:727063. doi: 10.3389/fvets.2021.727063 (PMC8761653; doi:10.3389/fvets.2021.727063)
Supplement: Supplementary file 1 [file Table_1.DOCX]

Supplementary table. Anaesthetic monitoring variables

|  | Mean +/- SD |
| --- | --- |
| HR (beats per minute) | 120 ± 19.4 |
| RR (breaths per minute) | 18 ± 4.9 |
| SpO_2_ (%) | 95.4 ± 1.66 |
| SAP (mmHg) | 115.9 ± 15.3 |
| DAP (mmHg) | 62.7 ± 14.5 |
| MAP (mmHg) | 80.7 ± 13.7 |

HR, Heart Rate; RR, Respiratory Rate; SpO_2_, pulse oximetry; SAP, Systolic Arterial Pressure, DAP, Diastolic Arterial Pressure, MAP, Mean Arterial Pressure
