# Supplementary material for: Rapid Patient-Side Evaluation of Endothelial Glycocalyx Thickness in Healthy Sedated Cats Using GlycoCheck® Software
Source: Front Vet Sci. 2022 Jan 3;8:727063. doi: 10.3389/fvets.2021.727063 (PMC8761653; doi:10.3389/fvets.2021.727063)
Supplement: Supplementary file 3 [file Data_Sheet_1.docx]

**Fit Group**

**Bivariate Fit of Ave vessel density By Age (months)**

**Linear Fit**

Ave vessel density = 167.33719 + 0.0565904*Age (months)

**Summary of Fit**

| RSquare | 0.0016 |
| --- | --- |
| RSquare Adj | -0.00869 |
| Root Mean Square Error | 65.82423 |
| Mean of Response | 171.1699 |
| Observations (or Sum Wgts) | 99 |

**Analysis of Variance**

| **Source** | **DF** | **Sum of Squares** | **Mean Square** | **F Ratio** |
| --- | --- | --- | --- | --- |
| Model | 1 | 673.65 | 673.65 | 0.1555 |
| Error | 97 | 420284.47 | 4332.83 | **Prob > F** |
| C. Total | 98 | 420958.12 |  | 0.6942 |

**Parameter Estimates**

| **Term** | **Estimate** | **Std Error** | **t Ratio** | **Prob>\|t\|** |
| --- | --- | --- | --- | --- |
| Intercept | 167.33719 | 11.75789 | 14.23 | <.0001* |
| Age (months) | 0.0565904 | 0.143519 | 0.39 | 0.6942 |

**Bivariate Fit of Ave vessel density By Weight (kg)**

**Linear Fit**

Ave vessel density = 120.85621 + 14.087966*Weight (kg)

**Summary of Fit**

| RSquare | 0.029314 |
| --- | --- |
| RSquare Adj | 0.019509 |
| Root Mean Square Error | 64.42348 |
| Mean of Response | 171.2688 |
| Observations (or Sum Wgts) | 101 |

**Analysis of Variance**

| **Source** | **DF** | **Sum of Squares** | **Mean Square** | **F Ratio** |
| --- | --- | --- | --- | --- |
| Model | 1 | 12408.62 | 12408.6 | 2.9898 |
| Error | 99 | 410888.03 | 4150.4 | **Prob > F** |
| C. Total | 100 | 423296.65 |  | 0.0869 |

**Parameter Estimates**

| **Term** | **Estimate** | **Std Error** | **t Ratio** | **Prob>\|t\|** |
| --- | --- | --- | --- | --- |
| Intercept | 120.85621 | 29.85197 | 4.05 | 0.0001* |
| Weight (kg) | 14.087966 | 8.147618 | 1.73 | 0.0869 |

**Bivariate Fit of Ave vessel density By HR (b/min)**

**Linear Fit**

Ave vessel density = 190.47295 - 0.1599388*HR (b/min)

**Summary of Fit**

| RSquare | 0.002269 |
| --- | --- |
| RSquare Adj | -0.00781 |
| Root Mean Square Error | 65.31478 |
| Mean of Response | 171.2688 |
| Observations (or Sum Wgts) | 101 |

**Analysis of Variance**

| **Source** | **DF** | **Sum of Squares** | **Mean Square** | **F Ratio** |
| --- | --- | --- | --- | --- |
| Model | 1 | 960.60 | 960.60 | 0.2252 |
| Error | 99 | 422336.05 | 4266.02 | **Prob > F** |
| C. Total | 100 | 423296.65 |  | 0.6362 |

**Parameter Estimates**

| **Term** | **Estimate** | **Std Error** | **t Ratio** | **Prob>\|t\|** |
| --- | --- | --- | --- | --- |
| Intercept | 190.47295 | 40.98866 | 4.65 | <.0001* |
| HR (b/min) | -0.159939 | 0.33705 | -0.47 | 0.6362 |

**Bivariate Fit of Ave vessel density By SpO2 (%)**

**Linear Fit**

Ave vessel density = 142.05379 + 0.3061228*SpO2 (%)

**Summary of Fit**

| RSquare | 0.000061 |
| --- | --- |
| RSquare Adj | -0.01004 |
| Root Mean Square Error | 65.38703 |
| Mean of Response | 171.2688 |
| Observations (or Sum Wgts) | 101 |

**Analysis of Variance**

| **Source** | **DF** | **Sum of Squares** | **Mean Square** | **F Ratio** |
| --- | --- | --- | --- | --- |
| Model | 1 | 25.78 | 25.78 | 0.0060 |
| Error | 99 | 423270.87 | 4275.46 | **Prob > F** |
| C. Total | 100 | 423296.65 |  | 0.9383 |

**Parameter Estimates**

| **Term** | **Estimate** | **Std Error** | **t Ratio** | **Prob>\|t\|** |
| --- | --- | --- | --- | --- |
| Intercept | 142.05379 | 376.2828 | 0.38 | 0.7066 |
| SpO2 (%) | 0.3061228 | 3.942202 | 0.08 | 0.9383 |

**Bivariate Fit of Ave vessel density By SAP (mmHg)**

**Linear Fit**

Ave vessel density = 183.61534 - 0.1065462*SAP (mmHg)

**Summary of Fit**

| RSquare | 0.000629 |
| --- | --- |
| RSquare Adj | -0.00947 |
| Root Mean Square Error | 65.36844 |
| Mean of Response | 171.2688 |
| Observations (or Sum Wgts) | 101 |

**Analysis of Variance**

| **Source** | **DF** | **Sum of Squares** | **Mean Square** | **F Ratio** |
| --- | --- | --- | --- | --- |
| Model | 1 | 266.40 | 266.40 | 0.0623 |
| Error | 99 | 423030.25 | 4273.03 | **Prob > F** |
| C. Total | 100 | 423296.65 |  | 0.8033 |

**Parameter Estimates**

| **Term** | **Estimate** | **Std Error** | **t Ratio** | **Prob>\|t\|** |
| --- | --- | --- | --- | --- |
| Intercept | 183.61534 | 49.87383 | 3.68 | 0.0004* |
| SAP (mmHg) | -0.106546 | 0.426718 | -0.25 | 0.8033 |

**Bivariate Fit of Ave vessel density By DAP (mmHg)**

**Linear Fit**

Ave vessel density = 145.99516 + 0.4027939*DAP (mmHg)

**Summary of Fit**

| RSquare | 0.00812 |
| --- | --- |
| RSquare Adj | -0.0019 |
| Root Mean Square Error | 65.12299 |
| Mean of Response | 171.2688 |
| Observations (or Sum Wgts) | 101 |

**Analysis of Variance**

| **Source** | **DF** | **Sum of Squares** | **Mean Square** | **F Ratio** |
| --- | --- | --- | --- | --- |
| Model | 1 | 3437.28 | 3437.28 | 0.8105 |
| Error | 99 | 419859.38 | 4241.00 | **Prob > F** |
| C. Total | 100 | 423296.65 |  | 0.3702 |

**Parameter Estimates**

| **Term** | **Estimate** | **Std Error** | **t Ratio** | **Prob>\|t\|** |
| --- | --- | --- | --- | --- |
| Intercept | 145.99516 | 28.81157 | 5.07 | <.0001* |
| DAP (mmHg) | 0.4027939 | 0.447415 | 0.90 | 0.3702 |

**Bivariate Fit of Ave vessel density By MAP (mmHg)**

**Linear Fit**

Ave vessel density = 162.88007 + 0.1040704*MAP (mmHg)

**Summary of Fit**

| RSquare | 0.000481 |
| --- | --- |
| RSquare Adj | -0.00961 |
| Root Mean Square Error | 65.37328 |
| Mean of Response | 171.2688 |
| Observations (or Sum Wgts) | 101 |

**Analysis of Variance**

| **Source** | **DF** | **Sum of Squares** | **Mean Square** | **F Ratio** |
| --- | --- | --- | --- | --- |
| Model | 1 | 203.69 | 203.69 | 0.0477 |
| Error | 99 | 423092.96 | 4273.67 | **Prob > F** |
| C. Total | 100 | 423296.65 |  | 0.8276 |

**Parameter Estimates**

| **Term** | **Estimate** | **Std Error** | **t Ratio** | **Prob>\|t\|** |
| --- | --- | --- | --- | --- |
| Intercept | 162.88007 | 38.97111 | 4.18 | <.0001* |
| MAP (mmHg) | 0.1040704 | 0.476691 | 0.22 | 0.8276 |

**Bivariate Fit of Ave vessel density By RR (bpm)**

**Linear Fit**

Ave vessel density = 144.52109 + 1.4201085*RR (bpm)

**Summary of Fit**

| RSquare | 0.011297 |
| --- | --- |
| RSquare Adj | 0.00131 |
| Root Mean Square Error | 65.01864 |
| Mean of Response | 171.2688 |
| Observations (or Sum Wgts) | 101 |

**Analysis of Variance**

| **Source** | **DF** | **Sum of Squares** | **Mean Square** | **F Ratio** |
| --- | --- | --- | --- | --- |
| Model | 1 | 4781.78 | 4781.78 | 1.1311 |
| Error | 99 | 418514.87 | 4227.42 | **Prob > F** |
| C. Total | 100 | 423296.65 |  | 0.2901 |

**Parameter Estimates**

| **Term** | **Estimate** | **Std Error** | **t Ratio** | **Prob>\|t\|** |
| --- | --- | --- | --- | --- |
| Intercept | 144.52109 | 25.96833 | 5.57 | <.0001* |
| RR (bpm) | 1.4201085 | 1.335256 | 1.06 | 0.2901 |

**Bivariate Fit of Ave vessel density By Temp ©**

**Linear Fit**

Ave vessel density = 182.91444 - 0.3074091*Temp ©

**Summary of Fit**

| RSquare | 8.219e-6 |
| --- | --- |
| RSquare Adj | -0.01009 |
| Root Mean Square Error | 65.38875 |
| Mean of Response | 171.2688 |
| Observations (or Sum Wgts) | 101 |

**Analysis of Variance**

| **Source** | **DF** | **Sum of Squares** | **Mean Square** | **F Ratio** |
| --- | --- | --- | --- | --- |
| Model | 1 | 3.48 | 3.48 | 0.0008 |
| Error | 99 | 423293.17 | 4275.69 | **Prob > F** |
| C. Total | 100 | 423296.65 |  | 0.9773 |

**Parameter Estimates**

| **Term** | **Estimate** | **Std Error** | **t Ratio** | **Prob>\|t\|** |
| --- | --- | --- | --- | --- |
| Intercept | 182.91444 | 408.3073 | 0.45 | 0.6551 |
| Temp © | -0.307409 | 10.7767 | -0.03 | 0.9773 |

**Oneway Analysis of Ave vessel density By Sex**

**Oneway Anova**

**Summary of Fit**

| Rsquare | 0.01976 |
| --- | --- |
| Adj Rsquare | -0.0112 |
| Root Mean Square Error | 65.90584 |
| Mean of Response | 171.1699 |
| Observations (or Sum Wgts) | 99 |

**Analysis of Variance**

| **Source** | **DF** | **Sum of Squares** | **Mean Square** | **F Ratio** | **Prob > F** |
| --- | --- | --- | --- | --- | --- |
| Sex | 3 | 8318.02 | 2772.67 | 0.6383 | 0.5922 |
| Error | 95 | 412640.11 | 4343.58 |  |  |
| C. Total | 98 | 420958.12 |  |  |  |

**Means and Std Deviations**

| **Level** | **Number** | **Mean** | **Std Dev** | **Std Err Mean** | **Lower 95%** | **Upper 95%** |
| --- | --- | --- | --- | --- | --- | --- |
| 0 | 35 | 172.55229 | 73.395394 | 12.406086 | 147.34009 | 197.76449 |
| 1 | 10 | 194.8 | 73.881447 | 23.363365 | 141.9484 | 247.6516 |
| 2 | 17 | 172.75471 | 62.413643 | 15.137532 | 140.66457 | 204.84484 |
| 3 | 37 | 162.74757 | 57.259748 | 9.4134446 | 143.65622 | 181.83892 |

**Means Comparisons**

**Comparisons for all pairs using Tukey-Kramer HSD**

**Connecting Letters Report**

| **Level** |  | **Mean** |
| --- | --- | --- |
| 1 | A | 194.80000 |
| 2 | A | 172.75471 |
| 0 | A | 172.55229 |
| 3 | A | 162.74757 |

Levels not connected by same letter are significantly different.

Missing Rows2**Oneway Analysis of Ave vessel density By Pre-med protocol**

**Oneway Anova**

**Summary of Fit**

| Rsquare | 0.000405 |
| --- | --- |
| Adj Rsquare | -0.00969 |
| Root Mean Square Error | 65.37579 |
| Mean of Response | 171.2688 |
| Observations (or Sum Wgts) | 101 |

**Analysis of Variance**

| **Source** | **DF** | **Sum of Squares** | **Mean Square** | **F Ratio** | **Prob > F** |
| --- | --- | --- | --- | --- | --- |
| Pre-med protocol | 1 | 171.26 | 171.26 | 0.0401 | 0.8418 |
| Error | 99 | 423125.39 | 4273.99 |  |  |
| C. Total | 100 | 423296.65 |  |  |  |

**Means and Std Deviations**

| **Level** | **Number** | **Mean** | **Std Dev** | **Std Err Mean** | **Lower 95%** | **Upper 95%** |
| --- | --- | --- | --- | --- | --- | --- |
| 1 | 12 | 167.7225 | 76.342994 | 22.038324 | 119.21648 | 216.22852 |
| 2 | 89 | 171.74697 | 63.872619 | 6.7704841 | 158.29205 | 185.20188 |

**Means Comparisons**

**Comparisons for all pairs using Tukey-Kramer HSD**

**Bivariate Fit of Ave RBC filling % By Age (months)**

**Linear Fit**

Ave RBC filling % = 77.139487 - 0.0602504*Age (months)

**Summary of Fit**

| RSquare | 0.222053 |
| --- | --- |
| RSquare Adj | 0.214033 |
| Root Mean Square Error | 5.251652 |
| Mean of Response | 73.05889 |
| Observations (or Sum Wgts) | 99 |

**Analysis of Variance**

| **Source** | **DF** | **Sum of Squares** | **Mean Square** | **F Ratio** |
| --- | --- | --- | --- | --- |
| Model | 1 | 763.6081 | 763.608 | 27.6872 |
| Error | 97 | 2675.2453 | 27.580 | **Prob > F** |
| C. Total | 98 | 3438.8534 |  | <.0001* |

**Parameter Estimates**

| **Term** | **Estimate** | **Std Error** | **t Ratio** | **Prob>\|t\|** |
| --- | --- | --- | --- | --- |
| Intercept | 77.139487 | 0.938079 | 82.23 | <.0001* |
| Age (months) | -0.06025 | 0.01145 | -5.26 | <.0001* |

**Bivariate Fit of Ave RBC filling % By Weight (kg)**

**Linear Fit**

Ave RBC filling % = 70.467774 + 0.7283626*Weight (kg)

**Summary of Fit**

| RSquare | 0.009457 |
| --- | --- |
| RSquare Adj | -0.00055 |
| Root Mean Square Error | 5.92387 |
| Mean of Response | 73.07416 |
| Observations (or Sum Wgts) | 101 |

**Analysis of Variance**

| **Source** | **DF** | **Sum of Squares** | **Mean Square** | **F Ratio** |
| --- | --- | --- | --- | --- |
| Model | 1 | 33.1682 | 33.1682 | 0.9452 |
| Error | 99 | 3474.1308 | 35.0922 | **Prob > F** |
| C. Total | 100 | 3507.2991 |  | 0.3333 |

**Parameter Estimates**

| **Term** | **Estimate** | **Std Error** | **t Ratio** | **Prob>\|t\|** |
| --- | --- | --- | --- | --- |
| Intercept | 70.467774 | 2.744949 | 25.67 | <.0001* |
| Weight (kg) | 0.7283626 | 0.74919 | 0.97 | 0.3333 |

**Bivariate Fit of Ave RBC filling % By HR (b/min)**

**Linear Fit**

Ave RBC filling % = 80.005461 - 0.0577263*HR (b/min)

**Summary of Fit**

| RSquare | 0.035679 |
| --- | --- |
| RSquare Adj | 0.025938 |
| Root Mean Square Error | 5.844935 |
| Mean of Response | 73.07416 |
| Observations (or Sum Wgts) | 101 |

**Analysis of Variance**

| **Source** | **DF** | **Sum of Squares** | **Mean Square** | **F Ratio** |
| --- | --- | --- | --- | --- |
| Model | 1 | 125.1363 | 125.136 | 3.6629 |
| Error | 99 | 3382.1628 | 34.163 | **Prob > F** |
| C. Total | 100 | 3507.2991 |  | 0.0585 |

**Parameter Estimates**

| **Term** | **Estimate** | **Std Error** | **t Ratio** | **Prob>\|t\|** |
| --- | --- | --- | --- | --- |
| Intercept | 80.005461 | 3.668021 | 21.81 | <.0001* |
| HR (b/min) | -0.057726 | 0.030162 | -1.91 | 0.0585 |

**Bivariate Fit of Ave RBC filling % By SpO2 (%)**

**Linear Fit**

Ave RBC filling % = 97.096901 - 0.2517167*SpO2 (%)

**Summary of Fit**

| RSquare | 0.00497 |
| --- | --- |
| RSquare Adj | -0.00508 |
| Root Mean Square Error | 5.937271 |
| Mean of Response | 73.07416 |
| Observations (or Sum Wgts) | 101 |

**Analysis of Variance**

| **Source** | **DF** | **Sum of Squares** | **Mean Square** | **F Ratio** |
| --- | --- | --- | --- | --- |
| Model | 1 | 17.4313 | 17.4313 | 0.4945 |
| Error | 99 | 3489.8678 | 35.2512 | **Prob > F** |
| C. Total | 100 | 3507.2991 |  | 0.4836 |

**Parameter Estimates**

| **Term** | **Estimate** | **Std Error** | **t Ratio** | **Prob>\|t\|** |
| --- | --- | --- | --- | --- |
| Intercept | 97.096901 | 34.16722 | 2.84 | 0.0054* |
| SpO2 (%) | -0.251717 | 0.35796 | -0.70 | 0.4836 |

**Bivariate Fit of Ave RBC filling % By SAP (mmHg)**

**Linear Fit**

Ave RBC filling % = 69.571582 + 0.030226*SAP (mmHg)

**Summary of Fit**

| RSquare | 0.006113 |
| --- | --- |
| RSquare Adj | -0.00393 |
| Root Mean Square Error | 5.933861 |
| Mean of Response | 73.07416 |
| Observations (or Sum Wgts) | 101 |

**Analysis of Variance**

| **Source** | **DF** | **Sum of Squares** | **Mean Square** | **F Ratio** |
| --- | --- | --- | --- | --- |
| Model | 1 | 21.4396 | 21.4396 | 0.6089 |
| Error | 99 | 3485.8595 | 35.2107 | **Prob > F** |
| C. Total | 100 | 3507.2991 |  | 0.4371 |

**Parameter Estimates**

| **Term** | **Estimate** | **Std Error** | **t Ratio** | **Prob>\|t\|** |
| --- | --- | --- | --- | --- |
| Intercept | 69.571582 | 4.527328 | 15.37 | <.0001* |
| SAP (mmHg) | 0.030226 | 0.038736 | 0.78 | 0.4371 |

**Bivariate Fit of Ave RBC filling % By DAP (mmHg)**

**Linear Fit**

Ave RBC filling % = 71.818484 + 0.0200121*DAP (mmHg)

**Summary of Fit**

| RSquare | 0.002419 |
| --- | --- |
| RSquare Adj | -0.00766 |
| Root Mean Square Error | 5.944877 |
| Mean of Response | 73.07416 |
| Observations (or Sum Wgts) | 101 |

**Analysis of Variance**

| **Source** | **DF** | **Sum of Squares** | **Mean Square** | **F Ratio** |
| --- | --- | --- | --- | --- |
| Model | 1 | 8.4846 | 8.4846 | 0.2401 |
| Error | 99 | 3498.8144 | 35.3416 | **Prob > F** |
| C. Total | 100 | 3507.2991 |  | 0.6252 |

**Parameter Estimates**

| **Term** | **Estimate** | **Std Error** | **t Ratio** | **Prob>\|t\|** |
| --- | --- | --- | --- | --- |
| Intercept | 71.818484 | 2.63012 | 27.31 | <.0001* |
| DAP (mmHg) | 0.0200121 | 0.040843 | 0.49 | 0.6252 |

**Bivariate Fit of Ave RBC filling % By MAP (mmHg)**

**Linear Fit**

Ave RBC filling % = 71.489129 + 0.0196638*MAP (mmHg)

**Summary of Fit**

| RSquare | 0.002073 |
| --- | --- |
| RSquare Adj | -0.00801 |
| Root Mean Square Error | 5.945907 |
| Mean of Response | 73.07416 |
| Observations (or Sum Wgts) | 101 |

**Analysis of Variance**

| **Source** | **DF** | **Sum of Squares** | **Mean Square** | **F Ratio** |
| --- | --- | --- | --- | --- |
| Model | 1 | 7.2721 | 7.2721 | 0.2057 |
| Error | 99 | 3500.0269 | 35.3538 | **Prob > F** |
| C. Total | 100 | 3507.2991 |  | 0.6512 |

**Parameter Estimates**

| **Term** | **Estimate** | **Std Error** | **t Ratio** | **Prob>\|t\|** |
| --- | --- | --- | --- | --- |
| Intercept | 71.489129 | 3.544546 | 20.17 | <.0001* |
| MAP (mmHg) | 0.0196638 | 0.043357 | 0.45 | 0.6512 |

**Bivariate Fit of Ave RBC filling % By RR (bpm)**

**Linear Fit**

Ave RBC filling % = 72.931417 + 0.0075785*RR (bpm)

**Summary of Fit**

| RSquare | 3.883e-5 |
| --- | --- |
| RSquare Adj | -0.01006 |
| Root Mean Square Error | 5.951965 |
| Mean of Response | 73.07416 |
| Observations (or Sum Wgts) | 101 |

**Analysis of Variance**

| **Source** | **DF** | **Sum of Squares** | **Mean Square** | **F Ratio** |
| --- | --- | --- | --- | --- |
| Model | 1 | 0.1362 | 0.1362 | 0.0038 |
| Error | 99 | 3507.1629 | 35.4259 | **Prob > F** |
| C. Total | 100 | 3507.2991 |  | 0.9507 |

**Parameter Estimates**

| **Term** | **Estimate** | **Std Error** | **t Ratio** | **Prob>\|t\|** |
| --- | --- | --- | --- | --- |
| Intercept | 72.931417 | 2.377204 | 30.68 | <.0001* |
| RR (bpm) | 0.0075785 | 0.122233 | 0.06 | 0.9507 |

**Bivariate Fit of Ave RBC filling % By Temp ©**

**Linear Fit**

Ave RBC filling % = 58.610757 + 0.3817896*Temp ©

**Summary of Fit**

| RSquare | 0.00153 |
| --- | --- |
| RSquare Adj | -0.00856 |
| Root Mean Square Error | 5.947525 |
| Mean of Response | 73.07416 |
| Observations (or Sum Wgts) | 101 |

**Analysis of Variance**

| **Source** | **DF** | **Sum of Squares** | **Mean Square** | **F Ratio** |
| --- | --- | --- | --- | --- |
| Model | 1 | 5.3664 | 5.3664 | 0.1517 |
| Error | 99 | 3501.9327 | 35.3731 | **Prob > F** |
| C. Total | 100 | 3507.2991 |  | 0.6977 |

**Parameter Estimates**

| **Term** | **Estimate** | **Std Error** | **t Ratio** | **Prob>\|t\|** |
| --- | --- | --- | --- | --- |
| Intercept | 58.610757 | 37.13816 | 1.58 | 0.1177 |
| Temp © | 0.3817896 | 0.98021 | 0.39 | 0.6977 |

**Oneway Analysis of Ave RBC filling % By Sex**

**Oneway Anova**

**Summary of Fit**

| Rsquare | 0.123439 |
| --- | --- |
| Adj Rsquare | 0.095758 |
| Root Mean Square Error | 5.632952 |
| Mean of Response | 73.05889 |
| Observations (or Sum Wgts) | 99 |

**Analysis of Variance**

| **Source** | **DF** | **Sum of Squares** | **Mean Square** | **F Ratio** | **Prob > F** |
| --- | --- | --- | --- | --- | --- |
| Sex | 3 | 424.4898 | 141.497 | 4.4594 | 0.0056* |
| Error | 95 | 3014.3636 | 31.730 |  |  |
| C. Total | 98 | 3438.8534 |  |  |  |

**Means and Std Deviations**

| **Level** | **Number** | **Mean** | **Std Dev** | **Std Err Mean** | **Lower 95%** | **Upper 95%** |
| --- | --- | --- | --- | --- | --- | --- |
| 0 | 35 | 72.475143 | 6.2313748 | 1.0532946 | 70.334591 | 74.615695 |
| 1 | 10 | 79.074 | 6.6468091 | 2.1019056 | 74.319159 | 83.828841 |
| 2 | 17 | 71.391765 | 6.1464596 | 1.4907354 | 68.231547 | 74.551983 |
| 3 | 37 | 72.751351 | 4.3845006 | 0.7208075 | 71.289486 | 74.213217 |

**Means Comparisons**

**Comparisons for all pairs using Tukey-Kramer HSD**

**Connecting Letters Report**

| **Level** |  |  | **Mean** |
| --- | --- | --- | --- |
| 1 | A |  | 79.074000 |
| 3 |  | B | 72.751351 |
| 0 |  | B | 72.475143 |
| 2 |  | B | 71.391765 |

Levels not connected by same letter are significantly different.

Missing Rows2**Oneway Analysis of Ave RBC filling % By Pre-med protocol**

**Oneway Anova**

**Summary of Fit**

| Rsquare | 0.020913 |
| --- | --- |
| Adj Rsquare | 0.011024 |
| Root Mean Square Error | 5.889513 |
| Mean of Response | 73.07416 |
| Observations (or Sum Wgts) | 101 |

**Analysis of Variance**

| **Source** | **DF** | **Sum of Squares** | **Mean Square** | **F Ratio** | **Prob > F** |
| --- | --- | --- | --- | --- | --- |
| Pre-med protocol | 1 | 73.3495 | 73.3495 | 2.1147 | 0.1491 |
| Error | 99 | 3433.9495 | 34.6864 |  |  |
| C. Total | 100 | 3507.2991 |  |  |  |

**Means and Std Deviations**

| **Level** | **Number** | **Mean** | **Std Dev** | **Std Err Mean** | **Lower 95%** | **Upper 95%** |
| --- | --- | --- | --- | --- | --- | --- |
| 1 | 12 | 70.753333 | 6.7298593 | 1.942743 | 66.477385 | 75.029282 |
| 2 | 89 | 73.387079 | 5.775879 | 0.6122419 | 72.170376 | 74.603781 |

**Means Comparisons**

**Comparisons for all pairs using Tukey-Kramer HSD**

**Bivariate Fit of Ave PBR 5-25 By Age (months)**

**Linear Fit**

Ave PBR 5-25 = 2.3192371 + 0.000833*Age (months)

**Summary of Fit**

| RSquare | 0.025439 |
| --- | --- |
| RSquare Adj | 0.015392 |
| Root Mean Square Error | 0.240109 |
| Mean of Response | 2.375657 |
| Observations (or Sum Wgts) | 99 |

**Analysis of Variance**

| **Source** | **DF** | **Sum of Squares** | **Mean Square** | **F Ratio** |
| --- | --- | --- | --- | --- |
| Model | 1 | 0.1459756 | 0.145976 | 2.5320 |
| Error | 97 | 5.5922567 | 0.057652 | **Prob > F** |
| C. Total | 98 | 5.7382323 |  | 0.1148 |

**Parameter Estimates**

| **Term** | **Estimate** | **Std Error** | **t Ratio** | **Prob>\|t\|** |
| --- | --- | --- | --- | --- |
| Intercept | 2.3192371 | 0.04289 | 54.07 | <.0001* |
| Age (months) | 0.000833 | 0.000524 | 1.59 | 0.1148 |

**Bivariate Fit of Ave PBR 5-25 By Weight (kg)**

**Linear Fit**

Ave PBR 5-25 = 2.4704427 - 0.0274603*Weight (kg)

**Summary of Fit**

| RSquare | 0.007869 |
| --- | --- |
| RSquare Adj | -0.00215 |
| Root Mean Square Error | 0.245027 |
| Mean of Response | 2.372178 |
| Observations (or Sum Wgts) | 101 |

**Analysis of Variance**

| **Source** | **DF** | **Sum of Squares** | **Mean Square** | **F Ratio** |
| --- | --- | --- | --- | --- |
| Model | 1 | 0.0471453 | 0.047145 | 0.7853 |
| Error | 99 | 5.9437755 | 0.060038 | **Prob > F** |
| C. Total | 100 | 5.9909208 |  | 0.3777 |

**Parameter Estimates**

| **Term** | **Estimate** | **Std Error** | **t Ratio** | **Prob>\|t\|** |
| --- | --- | --- | --- | --- |
| Intercept | 2.4704427 | 0.113538 | 21.76 | <.0001* |
| Weight (kg) | -0.02746 | 0.030988 | -0.89 | 0.3777 |

**Bivariate Fit of Ave PBR 5-25 By HR (b/min)**

**Linear Fit**

Ave PBR 5-25 = 2.1834612 + 0.0015717*HR (b/min)

**Summary of Fit**

| RSquare | 0.015484 |
| --- | --- |
| RSquare Adj | 0.005539 |
| Root Mean Square Error | 0.244085 |
| Mean of Response | 2.372178 |
| Observations (or Sum Wgts) | 101 |

**Analysis of Variance**

| **Source** | **DF** | **Sum of Squares** | **Mean Square** | **F Ratio** |
| --- | --- | --- | --- | --- |
| Model | 1 | 0.0927632 | 0.092763 | 1.5570 |
| Error | 99 | 5.8981576 | 0.059577 | **Prob > F** |
| C. Total | 100 | 5.9909208 |  | 0.2150 |

**Parameter Estimates**

| **Term** | **Estimate** | **Std Error** | **t Ratio** | **Prob>\|t\|** |
| --- | --- | --- | --- | --- |
| Intercept | 2.1834612 | 0.153177 | 14.25 | <.0001* |
| HR (b/min) | 0.0015717 | 0.00126 | 1.25 | 0.2150 |

**Bivariate Fit of Ave PBR 5-25 By SpO2 (%)**

**Linear Fit**

Ave PBR 5-25 = 0.820668 + 0.0162571*SpO2 (%)

**Summary of Fit**

| RSquare | 0.012137 |
| --- | --- |
| RSquare Adj | 0.002158 |
| Root Mean Square Error | 0.244499 |
| Mean of Response | 2.372178 |
| Observations (or Sum Wgts) | 101 |

**Analysis of Variance**

| **Source** | **DF** | **Sum of Squares** | **Mean Square** | **F Ratio** |
| --- | --- | --- | --- | --- |
| Model | 1 | 0.0727099 | 0.072710 | 1.2163 |
| Error | 99 | 5.9182109 | 0.059780 | **Prob > F** |
| C. Total | 100 | 5.9909208 |  | 0.2728 |

**Parameter Estimates**

| **Term** | **Estimate** | **Std Error** | **t Ratio** | **Prob>\|t\|** |
| --- | --- | --- | --- | --- |
| Intercept | 0.820668 | 1.40702 | 0.58 | 0.5610 |
| SpO2 (%) | 0.0162571 | 0.014741 | 1.10 | 0.2728 |

**Bivariate Fit of Ave PBR 5-25 By SAP (mmHg)**

**Linear Fit**

Ave PBR 5-25 = 2.7798119 - 0.0035177*SAP (mmHg)

**Summary of Fit**

| RSquare | 0.048472 |
| --- | --- |
| RSquare Adj | 0.03886 |
| Root Mean Square Error | 0.239961 |
| Mean of Response | 2.372178 |
| Observations (or Sum Wgts) | 101 |

**Analysis of Variance**

| **Source** | **DF** | **Sum of Squares** | **Mean Square** | **F Ratio** |
| --- | --- | --- | --- | --- |
| Model | 1 | 0.2903897 | 0.290390 | 5.0431 |
| Error | 99 | 5.7005310 | 0.057581 | **Prob > F** |
| C. Total | 100 | 5.9909208 |  | 0.0269* |

**Parameter Estimates**

| **Term** | **Estimate** | **Std Error** | **t Ratio** | **Prob>\|t\|** |
| --- | --- | --- | --- | --- |
| Intercept | 2.7798119 | 0.183082 | 15.18 | <.0001* |
| SAP (mmHg) | -0.003518 | 0.001566 | -2.25 | 0.0269* |

**Bivariate Fit of Ave PBR 5-25 By DAP (mmHg)**

**Linear Fit**

Ave PBR 5-25 = 2.5400726 - 0.0026758*DAP (mmHg)

**Summary of Fit**

| RSquare | 0.02532 |
| --- | --- |
| RSquare Adj | 0.015474 |
| Root Mean Square Error | 0.242862 |
| Mean of Response | 2.372178 |
| Observations (or Sum Wgts) | 101 |

**Analysis of Variance**

| **Source** | **DF** | **Sum of Squares** | **Mean Square** | **F Ratio** |
| --- | --- | --- | --- | --- |
| Model | 1 | 0.1516877 | 0.151688 | 2.5718 |
| Error | 99 | 5.8392330 | 0.058982 | **Prob > F** |
| C. Total | 100 | 5.9909208 |  | 0.1120 |

**Parameter Estimates**

| **Term** | **Estimate** | **Std Error** | **t Ratio** | **Prob>\|t\|** |
| --- | --- | --- | --- | --- |
| Intercept | 2.5400726 | 0.107447 | 23.64 | <.0001* |
| DAP (mmHg) | -0.002676 | 0.001669 | -1.60 | 0.1120 |

**Bivariate Fit of Ave PBR 5-25 By MAP (mmHg)**

**Linear Fit**

Ave PBR 5-25 = 2.650506 - 0.0034529*MAP (mmHg)

**Summary of Fit**

| RSquare | 0.037429 |
| --- | --- |
| RSquare Adj | 0.027706 |
| Root Mean Square Error | 0.241349 |
| Mean of Response | 2.372178 |
| Observations (or Sum Wgts) | 101 |

**Analysis of Variance**

| **Source** | **DF** | **Sum of Squares** | **Mean Square** | **F Ratio** |
| --- | --- | --- | --- | --- |
| Model | 1 | 0.2242331 | 0.224233 | 3.8495 |
| Error | 99 | 5.7666877 | 0.058249 | **Prob > F** |
| C. Total | 100 | 5.9909208 |  | 0.0526 |

**Parameter Estimates**

| **Term** | **Estimate** | **Std Error** | **t Ratio** | **Prob>\|t\|** |
| --- | --- | --- | --- | --- |
| Intercept | 2.650506 | 0.143876 | 18.42 | <.0001* |
| MAP (mmHg) | -0.003453 | 0.00176 | -1.96 | 0.0526 |

**Bivariate Fit of Ave PBR 5-25 By RR (bpm)**

**Linear Fit**

Ave PBR 5-25 = 2.3306492 + 0.0022049*RR (bpm)

**Summary of Fit**

| RSquare | 0.001924 |
| --- | --- |
| RSquare Adj | -0.00816 |
| Root Mean Square Error | 0.24576 |
| Mean of Response | 2.372178 |
| Observations (or Sum Wgts) | 101 |

**Analysis of Variance**

| **Source** | **DF** | **Sum of Squares** | **Mean Square** | **F Ratio** |
| --- | --- | --- | --- | --- |
| Model | 1 | 0.0115271 | 0.011527 | 0.1909 |
| Error | 99 | 5.9793937 | 0.060398 | **Prob > F** |
| C. Total | 100 | 5.9909208 |  | 0.6632 |

**Parameter Estimates**

| **Term** | **Estimate** | **Std Error** | **t Ratio** | **Prob>\|t\|** |
| --- | --- | --- | --- | --- |
| Intercept | 2.3306492 | 0.098156 | 23.74 | <.0001* |
| RR (bpm) | 0.0022049 | 0.005047 | 0.44 | 0.6632 |

**Bivariate Fit of Ave PBR 5-25 By Temp ©**

**Linear Fit**

Ave PBR 5-25 = 0.0262123 + 0.0619263*Temp ©

**Summary of Fit**

| RSquare | 0.023566 |
| --- | --- |
| RSquare Adj | 0.013703 |
| Root Mean Square Error | 0.243081 |
| Mean of Response | 2.372178 |
| Observations (or Sum Wgts) | 101 |

**Analysis of Variance**

| **Source** | **DF** | **Sum of Squares** | **Mean Square** | **F Ratio** |
| --- | --- | --- | --- | --- |
| Model | 1 | 0.1411840 | 0.141184 | 2.3894 |
| Error | 99 | 5.8497368 | 0.059088 | **Prob > F** |
| C. Total | 100 | 5.9909208 |  | 0.1254 |

**Parameter Estimates**

| **Term** | **Estimate** | **Std Error** | **t Ratio** | **Prob>\|t\|** |
| --- | --- | --- | --- | --- |
| Intercept | 0.0262123 | 1.51787 | 0.02 | 0.9863 |
| Temp © | 0.0619263 | 0.040062 | 1.55 | 0.1254 |

**Oneway Analysis of Ave PBR 5-25 By Sex**

**Oneway Anova**

**Summary of Fit**

| Rsquare | 0.089997 |
| --- | --- |
| Adj Rsquare | 0.06126 |
| Root Mean Square Error | 0.234449 |
| Mean of Response | 2.375657 |
| Observations (or Sum Wgts) | 99 |

**Analysis of Variance**

| **Source** | **DF** | **Sum of Squares** | **Mean Square** | **F Ratio** | **Prob > F** |
| --- | --- | --- | --- | --- | --- |
| Sex | 3 | 0.5164221 | 0.172141 | 3.1317 | 0.0292* |
| Error | 95 | 5.2218102 | 0.054966 |  |  |
| C. Total | 98 | 5.7382323 |  |  |  |

**Means and Std Deviations**

| **Level** | **Number** | **Mean** | **Std Dev** | **Std Err Mean** | **Lower 95%** | **Upper 95%** |
| --- | --- | --- | --- | --- | --- | --- |
| 0 | 35 | 2.4051429 | 0.2208056 | 0.037323 | 2.3292935 | 2.4809922 |
| 1 | 10 | 2.187 | 0.2321422 | 0.0734098 | 2.0209355 | 2.3530645 |
| 2 | 17 | 2.3229412 | 0.2404882 | 0.0583269 | 2.1992936 | 2.4465888 |
| 3 | 37 | 2.422973 | 0.2445956 | 0.0402113 | 2.3414207 | 2.5045252 |

**Means Comparisons**

**Comparisons for all pairs using Tukey-Kramer HSD**

**Connecting Letters Report**

| **Level** |  |  | **Mean** |
| --- | --- | --- | --- |
| 3 | A |  | 2.4229730 |
| 0 | A | B | 2.4051429 |
| 2 | A | B | 2.3229412 |
| 1 |  | B | 2.1870000 |

Levels not connected by same letter are significantly different.

Missing Rows2**Oneway Analysis of Ave PBR 5-25 By Pre-med protocol**

**Oneway Anova**

**Summary of Fit**

| Rsquare | 0.017532 |
| --- | --- |
| Adj Rsquare | 0.007608 |
| Root Mean Square Error | 0.243831 |
| Mean of Response | 2.372178 |
| Observations (or Sum Wgts) | 101 |

**Analysis of Variance**

| **Source** | **DF** | **Sum of Squares** | **Mean Square** | **F Ratio** | **Prob > F** |
| --- | --- | --- | --- | --- | --- |
| Pre-med protocol | 1 | 0.1050309 | 0.105031 | 1.7666 | 0.1869 |
| Error | 99 | 5.8858899 | 0.059453 |  |  |
| C. Total | 100 | 5.9909208 |  |  |  |

**Means and Std Deviations**

| **Level** | **Number** | **Mean** | **Std Dev** | **Std Err Mean** | **Lower 95%** | **Upper 95%** |
| --- | --- | --- | --- | --- | --- | --- |
| 1 | 12 | 2.46 | 0.3279412 | 0.0946685 | 2.2516361 | 2.6683639 |
| 2 | 89 | 2.3603371 | 0.2311751 | 0.0245045 | 2.3116395 | 2.4090346 |

**Means Comparisons**

**Comparisons for all pairs using Tukey-Kramer HSD**

**Bivariate Fit of Ave PBR 5-9 By Age (months)**

**Linear Fit**

Ave PBR 5-9 = 1.2901444 + 0.0008017*Age (months)

**Summary of Fit**

| RSquare | 0.063725 |
| --- | --- |
| RSquare Adj | 0.054073 |
| Root Mean Square Error | 0.143111 |
| Mean of Response | 1.344444 |
| Observations (or Sum Wgts) | 99 |

**Analysis of Variance**

| **Source** | **DF** | **Sum of Squares** | **Mean Square** | **F Ratio** |
| --- | --- | --- | --- | --- |
| Model | 1 | 0.1352143 | 0.135214 | 6.6020 |
| Error | 97 | 1.9866301 | 0.020481 | **Prob > F** |
| C. Total | 98 | 2.1218444 |  | 0.0117* |

**Parameter Estimates**

| **Term** | **Estimate** | **Std Error** | **t Ratio** | **Prob>\|t\|** |
| --- | --- | --- | --- | --- |
| Intercept | 1.2901444 | 0.025563 | 50.47 | <.0001* |
| Age (months) | 0.0008017 | 0.000312 | 2.57 | 0.0117* |

**Bivariate Fit of Ave PBR 5-9 By Weight (kg)**

**Linear Fit**

Ave PBR 5-9 = 1.3034502 + 0.0120124*Weight (kg)

**Summary of Fit**

| RSquare | 0.004184 |
| --- | --- |
| RSquare Adj | -0.00587 |
| Root Mean Square Error | 0.147268 |
| Mean of Response | 1.346436 |
| Observations (or Sum Wgts) | 101 |

**Analysis of Variance**

| **Source** | **DF** | **Sum of Squares** | **Mean Square** | **F Ratio** |
| --- | --- | --- | --- | --- |
| Model | 1 | 0.0090217 | 0.009022 | 0.4160 |
| Error | 99 | 2.1470952 | 0.021688 | **Prob > F** |
| C. Total | 100 | 2.1561168 |  | 0.5204 |

**Parameter Estimates**

| **Term** | **Estimate** | **Std Error** | **t Ratio** | **Prob>\|t\|** |
| --- | --- | --- | --- | --- |
| Intercept | 1.3034502 | 0.06824 | 19.10 | <.0001* |
| Weight (kg) | 0.0120124 | 0.018625 | 0.64 | 0.5204 |

**Bivariate Fit of Ave PBR 5-9 By HR (b/min)**

**Linear Fit**

Ave PBR 5-9 = 1.2103729 + 0.0011332*HR (b/min)

**Summary of Fit**

| RSquare | 0.022365 |
| --- | --- |
| RSquare Adj | 0.012489 |
| Root Mean Square Error | 0.145917 |
| Mean of Response | 1.346436 |
| Observations (or Sum Wgts) | 101 |

**Analysis of Variance**

| **Source** | **DF** | **Sum of Squares** | **Mean Square** | **F Ratio** |
| --- | --- | --- | --- | --- |
| Model | 1 | 0.0482205 | 0.048221 | 2.2647 |
| Error | 99 | 2.1078963 | 0.021292 | **Prob > F** |
| C. Total | 100 | 2.1561168 |  | 0.1355 |

**Parameter Estimates**

| **Term** | **Estimate** | **Std Error** | **t Ratio** | **Prob>\|t\|** |
| --- | --- | --- | --- | --- |
| Intercept | 1.2103729 | 0.091571 | 13.22 | <.0001* |
| HR (b/min) | 0.0011332 | 0.000753 | 1.50 | 0.1355 |

**Bivariate Fit of Ave PBR 5-9 By SpO2 (%)**

**Linear Fit**

Ave PBR 5-9 = 1.1821788 + 0.0017211*SpO2 (%)

**Summary of Fit**

| RSquare | 0.000378 |
| --- | --- |
| RSquare Adj | -0.00972 |
| Root Mean Square Error | 0.147549 |
| Mean of Response | 1.346436 |
| Observations (or Sum Wgts) | 101 |

**Analysis of Variance**

| **Source** | **DF** | **Sum of Squares** | **Mean Square** | **F Ratio** |
| --- | --- | --- | --- | --- |
| Model | 1 | 0.0008150 | 0.000815 | 0.0374 |
| Error | 99 | 2.1553019 | 0.021771 | **Prob > F** |
| C. Total | 100 | 2.1561168 |  | 0.8470 |

**Parameter Estimates**

| **Term** | **Estimate** | **Std Error** | **t Ratio** | **Prob>\|t\|** |
| --- | --- | --- | --- | --- |
| Intercept | 1.1821788 | 0.849101 | 1.39 | 0.1670 |
| SpO2 (%) | 0.0017211 | 0.008896 | 0.19 | 0.8470 |

**Bivariate Fit of Ave PBR 5-9 By SAP (mmHg)**

**Linear Fit**

Ave PBR 5-9 = 1.5489848 - 0.0017479*SAP (mmHg)

**Summary of Fit**

| RSquare | 0.033253 |
| --- | --- |
| RSquare Adj | 0.023488 |
| Root Mean Square Error | 0.145103 |
| Mean of Response | 1.346436 |
| Observations (or Sum Wgts) | 101 |

**Analysis of Variance**

| **Source** | **DF** | **Sum of Squares** | **Mean Square** | **F Ratio** |
| --- | --- | --- | --- | --- |
| Model | 1 | 0.0716972 | 0.071697 | 3.4053 |
| Error | 99 | 2.0844196 | 0.021055 | **Prob > F** |
| C. Total | 100 | 2.1561168 |  | 0.0680 |

**Parameter Estimates**

| **Term** | **Estimate** | **Std Error** | **t Ratio** | **Prob>\|t\|** |
| --- | --- | --- | --- | --- |
| Intercept | 1.5489848 | 0.110708 | 13.99 | <.0001* |
| SAP (mmHg) | -0.001748 | 0.000947 | -1.85 | 0.0680 |

**Bivariate Fit of Ave PBR 5-9 By DAP (mmHg)**

**Linear Fit**

Ave PBR 5-9 = 1.4779382 - 0.0020958*DAP (mmHg)

**Summary of Fit**

| RSquare | 0.043159 |
| --- | --- |
| RSquare Adj | 0.033494 |
| Root Mean Square Error | 0.144357 |
| Mean of Response | 1.346436 |
| Observations (or Sum Wgts) | 101 |

**Analysis of Variance**

| **Source** | **DF** | **Sum of Squares** | **Mean Square** | **F Ratio** |
| --- | --- | --- | --- | --- |
| Model | 1 | 0.0930565 | 0.093056 | 4.4655 |
| Error | 99 | 2.0630604 | 0.020839 | **Prob > F** |
| C. Total | 100 | 2.1561168 |  | 0.0371* |

**Parameter Estimates**

| **Term** | **Estimate** | **Std Error** | **t Ratio** | **Prob>\|t\|** |
| --- | --- | --- | --- | --- |
| Intercept | 1.4779382 | 0.063866 | 23.14 | <.0001* |
| DAP (mmHg) | -0.002096 | 0.000992 | -2.11 | 0.0371* |

**Bivariate Fit of Ave PBR 5-9 By MAP (mmHg)**

**Linear Fit**

Ave PBR 5-9 = 1.5355411 - 0.002346*MAP (mmHg)

**Summary of Fit**

| RSquare | 0.048009 |
| --- | --- |
| RSquare Adj | 0.038393 |
| Root Mean Square Error | 0.143991 |
| Mean of Response | 1.346436 |
| Observations (or Sum Wgts) | 101 |

**Analysis of Variance**

| **Source** | **DF** | **Sum of Squares** | **Mean Square** | **F Ratio** |
| --- | --- | --- | --- | --- |
| Model | 1 | 0.1035129 | 0.103513 | 4.9926 |
| Error | 99 | 2.0526039 | 0.020733 | **Prob > F** |
| C. Total | 100 | 2.1561168 |  | 0.0277* |

**Parameter Estimates**

| **Term** | **Estimate** | **Std Error** | **t Ratio** | **Prob>\|t\|** |
| --- | --- | --- | --- | --- |
| Intercept | 1.5355411 | 0.085838 | 17.89 | <.0001* |
| MAP (mmHg) | -0.002346 | 0.00105 | -2.23 | 0.0277* |

**Bivariate Fit of Ave PBR 5-9 By RR (bpm)**

**Linear Fit**

Ave PBR 5-9 = 1.3509853 - 0.0002416*RR (bpm)

**Summary of Fit**

| RSquare | 6.416e-5 |
| --- | --- |
| RSquare Adj | -0.01004 |
| Root Mean Square Error | 0.147572 |
| Mean of Response | 1.346436 |
| Observations (or Sum Wgts) | 101 |

**Analysis of Variance**

| **Source** | **DF** | **Sum of Squares** | **Mean Square** | **F Ratio** |
| --- | --- | --- | --- | --- |
| Model | 1 | 0.0001383 | 0.000138 | 0.0064 |
| Error | 99 | 2.1559785 | 0.021778 | **Prob > F** |
| C. Total | 100 | 2.1561168 |  | 0.9366 |

**Parameter Estimates**

| **Term** | **Estimate** | **Std Error** | **t Ratio** | **Prob>\|t\|** |
| --- | --- | --- | --- | --- |
| Intercept | 1.3509853 | 0.05894 | 22.92 | <.0001* |
| RR (bpm) | -0.000242 | 0.003031 | -0.08 | 0.9366 |

**Bivariate Fit of Ave PBR 5-9 By Temp ©**

**Linear Fit**

Ave PBR 5-9 = 2.0098531 - 0.0175122*Temp ©

**Summary of Fit**

| RSquare | 0.005237 |
| --- | --- |
| RSquare Adj | -0.00481 |
| Root Mean Square Error | 0.14719 |
| Mean of Response | 1.346436 |
| Observations (or Sum Wgts) | 101 |

**Analysis of Variance**

| **Source** | **DF** | **Sum of Squares** | **Mean Square** | **F Ratio** |
| --- | --- | --- | --- | --- |
| Model | 1 | 0.0112906 | 0.011291 | 0.5211 |
| Error | 99 | 2.1448263 | 0.021665 | **Prob > F** |
| C. Total | 100 | 2.1561168 |  | 0.4721 |

**Parameter Estimates**

| **Term** | **Estimate** | **Std Error** | **t Ratio** | **Prob>\|t\|** |
| --- | --- | --- | --- | --- |
| Intercept | 2.0098531 | 0.9191 | 2.19 | 0.0311* |
| Temp © | -0.017512 | 0.024258 | -0.72 | 0.4721 |

**Oneway Analysis of Ave PBR 5-9 By Sex**

**Oneway Anova**

**Summary of Fit**

| Rsquare | 0.115844 |
| --- | --- |
| Adj Rsquare | 0.087923 |
| Root Mean Square Error | 0.140527 |
| Mean of Response | 1.344444 |
| Observations (or Sum Wgts) | 99 |

**Analysis of Variance**

| **Source** | **DF** | **Sum of Squares** | **Mean Square** | **F Ratio** | **Prob > F** |
| --- | --- | --- | --- | --- | --- |
| Sex | 3 | 0.2458019 | 0.081934 | 4.1490 | 0.0083* |
| Error | 95 | 1.8760425 | 0.019748 |  |  |
| C. Total | 98 | 2.1218444 |  |  |  |

**Means and Std Deviations**

| **Level** | **Number** | **Mean** | **Std Dev** | **Std Err Mean** | **Lower 95%** | **Upper 95%** |
| --- | --- | --- | --- | --- | --- | --- |
| 0 | 35 | 1.3822857 | 0.1538732 | 0.0260093 | 1.3294284 | 1.435143 |
| 1 | 10 | 1.206 | 0.1571411 | 0.0496924 | 1.093588 | 1.318412 |
| 2 | 17 | 1.3329412 | 0.1373301 | 0.0333074 | 1.2623325 | 1.4035498 |
| 3 | 37 | 1.3513514 | 0.1232694 | 0.0202654 | 1.3102513 | 1.3924514 |

**Means Comparisons**

**Comparisons for all pairs using Tukey-Kramer HSD**

**Connecting Letters Report**

| **Level** |  |  | **Mean** |
| --- | --- | --- | --- |
| 0 | A |  | 1.3822857 |
| 3 | A |  | 1.3513514 |
| 2 | A | B | 1.3329412 |
| 1 |  | B | 1.2060000 |

Levels not connected by same letter are significantly different.

Missing Rows2**Oneway Analysis of Ave PBR 5-9 By Pre-med protocol**

**Oneway Anova**

**Summary of Fit**

| Rsquare | 0.001706 |
| --- | --- |
| Adj Rsquare | -0.00838 |
| Root Mean Square Error | 0.147451 |
| Mean of Response | 1.346436 |
| Observations (or Sum Wgts) | 101 |

**Analysis of Variance**

| **Source** | **DF** | **Sum of Squares** | **Mean Square** | **F Ratio** | **Prob > F** |
| --- | --- | --- | --- | --- | --- |
| Pre-med protocol | 1 | 0.0036786 | 0.003679 | 0.1692 | 0.6817 |
| Error | 99 | 2.1524382 | 0.021742 |  |  |
| C. Total | 100 | 2.1561168 |  |  |  |

**Means and Std Deviations**

| **Level** | **Number** | **Mean** | **Std Dev** | **Std Err Mean** | **Lower 95%** | **Upper 95%** |
| --- | --- | --- | --- | --- | --- | --- |
| 1 | 12 | 1.33 | 0.1561468 | 0.0450757 | 1.2307891 | 1.4292109 |
| 2 | 89 | 1.3486517 | 0.1463277 | 0.0155107 | 1.3178274 | 1.379476 |

**Means Comparisons**

**Comparisons for all pairs using Tukey-Kramer HSD**

**Bivariate Fit of Ave PBR 10-19 By Age (months)**

**Linear Fit**

Ave PBR 10-19 = 2.6428889 + 0.000797*Age (months)

**Summary of Fit**

| RSquare | 0.015859 |
| --- | --- |
| RSquare Adj | 0.005714 |
| Root Mean Square Error | 0.292376 |
| Mean of Response | 2.696869 |
| Observations (or Sum Wgts) | 99 |

**Analysis of Variance**

| **Source** | **DF** | **Sum of Squares** | **Mean Square** | **F Ratio** |
| --- | --- | --- | --- | --- |
| Model | 1 | 0.1336243 | 0.133624 | 1.5632 |
| Error | 97 | 8.2919050 | 0.085484 | **Prob > F** |
| C. Total | 98 | 8.4255293 |  | 0.2142 |

**Parameter Estimates**

| **Term** | **Estimate** | **Std Error** | **t Ratio** | **Prob>\|t\|** |
| --- | --- | --- | --- | --- |
| Intercept | 2.6428889 | 0.052226 | 50.61 | <.0001* |
| Age (months) | 0.000797 | 0.000637 | 1.25 | 0.2142 |

**Bivariate Fit of Ave PBR 10-19 By Weight (kg)**

**Linear Fit**

Ave PBR 10-19 = 2.8678925 - 0.0487442*Weight (kg)

**Summary of Fit**

| RSquare | 0.017005 |
| --- | --- |
| RSquare Adj | 0.007076 |
| Root Mean Square Error | 0.294511 |
| Mean of Response | 2.693465 |
| Observations (or Sum Wgts) | 101 |

**Analysis of Variance**

| **Source** | **DF** | **Sum of Squares** | **Mean Square** | **F Ratio** |
| --- | --- | --- | --- | --- |
| Model | 1 | 0.1485503 | 0.148550 | 1.7127 |
| Error | 99 | 8.5869368 | 0.086737 | **Prob > F** |
| C. Total | 100 | 8.7354871 |  | 0.1937 |

**Parameter Estimates**

| **Term** | **Estimate** | **Std Error** | **t Ratio** | **Prob>\|t\|** |
| --- | --- | --- | --- | --- |
| Intercept | 2.8678925 | 0.136468 | 21.02 | <.0001* |
| Weight (kg) | -0.048744 | 0.037247 | -1.31 | 0.1937 |

**Bivariate Fit of Ave PBR 10-19 By HR (b/min)**

**Linear Fit**

Ave PBR 10-19 = 2.367639 + 0.0027136*HR (b/min)

**Summary of Fit**

| RSquare | 0.031655 |
| --- | --- |
| RSquare Adj | 0.021873 |
| Root Mean Square Error | 0.292308 |
| Mean of Response | 2.693465 |
| Observations (or Sum Wgts) | 101 |

**Analysis of Variance**

| **Source** | **DF** | **Sum of Squares** | **Mean Square** | **F Ratio** |
| --- | --- | --- | --- | --- |
| Model | 1 | 0.2765197 | 0.276520 | 3.2363 |
| Error | 99 | 8.4589675 | 0.085444 | **Prob > F** |
| C. Total | 100 | 8.7354871 |  | 0.0751 |

**Parameter Estimates**

| **Term** | **Estimate** | **Std Error** | **t Ratio** | **Prob>\|t\|** |
| --- | --- | --- | --- | --- |
| Intercept | 2.367639 | 0.18344 | 12.91 | <.0001* |
| HR (b/min) | 0.0027136 | 0.001508 | 1.80 | 0.0751 |

**Bivariate Fit of Ave PBR 10-19 By SpO2 (%)**

**Linear Fit**

Ave PBR 10-19 = 1.29458 + 0.0146579*SpO2 (%)

**Summary of Fit**

| RSquare | 0.006766 |
| --- | --- |
| RSquare Adj | -0.00327 |
| Root Mean Square Error | 0.296041 |
| Mean of Response | 2.693465 |
| Observations (or Sum Wgts) | 101 |

**Analysis of Variance**

| **Source** | **DF** | **Sum of Squares** | **Mean Square** | **F Ratio** |
| --- | --- | --- | --- | --- |
| Model | 1 | 0.0591083 | 0.059108 | 0.6744 |
| Error | 99 | 8.6763788 | 0.087640 | **Prob > F** |
| C. Total | 100 | 8.7354871 |  | 0.4135 |

**Parameter Estimates**

| **Term** | **Estimate** | **Std Error** | **t Ratio** | **Prob>\|t\|** |
| --- | --- | --- | --- | --- |
| Intercept | 1.29458 | 1.703627 | 0.76 | 0.4491 |
| SpO2 (%) | 0.0146579 | 0.017848 | 0.82 | 0.4135 |

**Bivariate Fit of Ave PBR 10-19 By SAP (mmHg)**

**Linear Fit**

Ave PBR 10-19 = 3.1882352 - 0.0042697*SAP (mmHg)

**Summary of Fit**

| RSquare | 0.048973 |
| --- | --- |
| RSquare Adj | 0.039367 |
| Root Mean Square Error | 0.289683 |
| Mean of Response | 2.693465 |
| Observations (or Sum Wgts) | 101 |

**Analysis of Variance**

| **Source** | **DF** | **Sum of Squares** | **Mean Square** | **F Ratio** |
| --- | --- | --- | --- | --- |
| Model | 1 | 0.4278068 | 0.427807 | 5.0980 |
| Error | 99 | 8.3076804 | 0.083916 | **Prob > F** |
| C. Total | 100 | 8.7354871 |  | 0.0261* |

**Parameter Estimates**

| **Term** | **Estimate** | **Std Error** | **t Ratio** | **Prob>\|t\|** |
| --- | --- | --- | --- | --- |
| Intercept | 3.1882352 | 0.221018 | 14.43 | <.0001* |
| SAP (mmHg) | -0.00427 | 0.001891 | -2.26 | 0.0261* |

**Bivariate Fit of Ave PBR 10-19 By DAP (mmHg)**

**Linear Fit**

Ave PBR 10-19 = 2.9037717 - 0.0033517*DAP (mmHg)

**Summary of Fit**

| RSquare | 0.027246 |
| --- | --- |
| RSquare Adj | 0.01742 |
| Root Mean Square Error | 0.292973 |
| Mean of Response | 2.693465 |
| Observations (or Sum Wgts) | 101 |

**Analysis of Variance**

| **Source** | **DF** | **Sum of Squares** | **Mean Square** | **F Ratio** |
| --- | --- | --- | --- | --- |
| Model | 1 | 0.2380033 | 0.238003 | 2.7729 |
| Error | 99 | 8.4974838 | 0.085833 | **Prob > F** |
| C. Total | 100 | 8.7354871 |  | 0.0990 |

**Parameter Estimates**

| **Term** | **Estimate** | **Std Error** | **t Ratio** | **Prob>\|t\|** |
| --- | --- | --- | --- | --- |
| Intercept | 2.9037717 | 0.129616 | 22.40 | <.0001* |
| DAP (mmHg) | -0.003352 | 0.002013 | -1.67 | 0.0990 |

**Bivariate Fit of Ave PBR 10-19 By MAP (mmHg)**

**Linear Fit**

Ave PBR 10-19 = 3.0320767 - 0.0042008*MAP (mmHg)

**Summary of Fit**

| RSquare | 0.037993 |
| --- | --- |
| RSquare Adj | 0.028276 |
| Root Mean Square Error | 0.29135 |
| Mean of Response | 2.693465 |
| Observations (or Sum Wgts) | 101 |

**Analysis of Variance**

| **Source** | **DF** | **Sum of Squares** | **Mean Square** | **F Ratio** |
| --- | --- | --- | --- | --- |
| Model | 1 | 0.3318865 | 0.331887 | 3.9098 |
| Error | 99 | 8.4036006 | 0.084885 | **Prob > F** |
| C. Total | 100 | 8.7354871 |  | 0.0508 |

**Parameter Estimates**

| **Term** | **Estimate** | **Std Error** | **t Ratio** | **Prob>\|t\|** |
| --- | --- | --- | --- | --- |
| Intercept | 3.0320767 | 0.173683 | 17.46 | <.0001* |
| MAP (mmHg) | -0.004201 | 0.002124 | -1.98 | 0.0508 |

**Bivariate Fit of Ave PBR 10-19 By RR (bpm)**

**Linear Fit**

Ave PBR 10-19 = 2.6371364 + 0.0029907*RR (bpm)

**Summary of Fit**

| RSquare | 0.002428 |
| --- | --- |
| RSquare Adj | -0.00765 |
| Root Mean Square Error | 0.296687 |
| Mean of Response | 2.693465 |
| Observations (or Sum Wgts) | 101 |

**Analysis of Variance**

| **Source** | **DF** | **Sum of Squares** | **Mean Square** | **F Ratio** |
| --- | --- | --- | --- | --- |
| Model | 1 | 0.0212070 | 0.021207 | 0.2409 |
| Error | 99 | 8.7142801 | 0.088023 | **Prob > F** |
| C. Total | 100 | 8.7354871 |  | 0.6246 |

**Parameter Estimates**

| **Term** | **Estimate** | **Std Error** | **t Ratio** | **Prob>\|t\|** |
| --- | --- | --- | --- | --- |
| Intercept | 2.6371364 | 0.118496 | 22.26 | <.0001* |
| RR (bpm) | 0.0029907 | 0.006093 | 0.49 | 0.6246 |

**Bivariate Fit of Ave PBR 10-19 By Temp ©**

**Linear Fit**

Ave PBR 10-19 = -1.039603 + 0.0985416*Temp ©

**Summary of Fit**

| RSquare | 0.040925 |
| --- | --- |
| RSquare Adj | 0.031237 |
| Root Mean Square Error | 0.290906 |
| Mean of Response | 2.693465 |
| Observations (or Sum Wgts) | 101 |

**Analysis of Variance**

| **Source** | **DF** | **Sum of Squares** | **Mean Square** | **F Ratio** |
| --- | --- | --- | --- | --- |
| Model | 1 | 0.3574982 | 0.357498 | 4.2244 |
| Error | 99 | 8.3779889 | 0.084626 | **Prob > F** |
| C. Total | 100 | 8.7354871 |  | 0.0425* |

**Parameter Estimates**

| **Term** | **Estimate** | **Std Error** | **t Ratio** | **Prob>\|t\|** |
| --- | --- | --- | --- | --- |
| Intercept | -1.039603 | 1.816504 | -0.57 | 0.5684 |
| Temp © | 0.0985416 | 0.047944 | 2.06 | 0.0425* |

**Oneway Analysis of Ave PBR 10-19 By Sex**

**Oneway Anova**

**Summary of Fit**

| Rsquare | 0.103891 |
| --- | --- |
| Adj Rsquare | 0.075592 |
| Root Mean Square Error | 0.281914 |
| Mean of Response | 2.696869 |
| Observations (or Sum Wgts) | 99 |

**Analysis of Variance**

| **Source** | **DF** | **Sum of Squares** | **Mean Square** | **F Ratio** | **Prob > F** |
| --- | --- | --- | --- | --- | --- |
| Sex | 3 | 0.8753336 | 0.291778 | 3.6713 | 0.0149* |
| Error | 95 | 7.5501957 | 0.079476 |  |  |
| C. Total | 98 | 8.4255293 |  |  |  |

**Means and Std Deviations**

| **Level** | **Number** | **Mean** | **Std Dev** | **Std Err Mean** | **Lower 95%** | **Upper 95%** |
| --- | --- | --- | --- | --- | --- | --- |
| 0 | 35 | 2.7197143 | 0.2939536 | 0.0496872 | 2.6187377 | 2.8206909 |
| 1 | 10 | 2.431 | 0.2218834 | 0.0701657 | 2.2722742 | 2.5897258 |
| 2 | 17 | 2.6723529 | 0.2353595 | 0.0570831 | 2.5513422 | 2.7933637 |
| 3 | 37 | 2.7583784 | 0.3019797 | 0.0496452 | 2.6576933 | 2.8590634 |

**Means Comparisons**

**Comparisons for all pairs using Tukey-Kramer HSD**

**Connecting Letters Report**

| **Level** |  |  | **Mean** |
| --- | --- | --- | --- |
| 3 | A |  | 2.7583784 |
| 0 | A |  | 2.7197143 |
| 2 | A | B | 2.6723529 |
| 1 |  | B | 2.4310000 |

Levels not connected by same letter are significantly different.

Missing Rows2**Oneway Analysis of Ave PBR 10-19 By Pre-med protocol**

**Oneway Anova**

**Summary of Fit**

| Rsquare | 0.021475 |
| --- | --- |
| Adj Rsquare | 0.01159 |
| Root Mean Square Error | 0.293841 |
| Mean of Response | 2.693465 |
| Observations (or Sum Wgts) | 101 |

**Analysis of Variance**

| **Source** | **DF** | **Sum of Squares** | **Mean Square** | **F Ratio** | **Prob > F** |
| --- | --- | --- | --- | --- | --- |
| Pre-med protocol | 1 | 0.1875910 | 0.187591 | 2.1726 | 0.1437 |
| Error | 99 | 8.5478962 | 0.086342 |  |  |
| C. Total | 100 | 8.7354871 |  |  |  |

**Means and Std Deviations**

| **Level** | **Number** | **Mean** | **Std Dev** | **Std Err Mean** | **Lower 95%** | **Upper 95%** |
| --- | --- | --- | --- | --- | --- | --- |
| 1 | 12 | 2.8108333 | 0.3937802 | 0.1136746 | 2.5606373 | 3.0610294 |
| 2 | 89 | 2.6776404 | 0.278841 | 0.0295571 | 2.6189019 | 2.736379 |

**Means Comparisons**

**Comparisons for all pairs using Tukey-Kramer HSD**

**Bivariate Fit of Ave PBR 20-25 By Age (months)**

**Linear Fit**

Ave PBR 20-25 = 2.7628391 + 0.0023578*Age (months)

**Summary of Fit**

| RSquare | 0.045861 |
| --- | --- |
| RSquare Adj | 0.036024 |
| Root Mean Square Error | 0.500817 |
| Mean of Response | 2.922525 |
| Observations (or Sum Wgts) | 99 |

**Analysis of Variance**

| **Source** | **DF** | **Sum of Squares** | **Mean Square** | **F Ratio** |
| --- | --- | --- | --- | --- |
| Model | 1 | 1.169385 | 1.16939 | 4.6623 |
| Error | 97 | 24.329283 | 0.25082 | **Prob > F** |
| C. Total | 98 | 25.498669 |  | 0.0333* |

**Parameter Estimates**

| **Term** | **Estimate** | **Std Error** | **t Ratio** | **Prob>\|t\|** |
| --- | --- | --- | --- | --- |
| Intercept | 2.7628391 | 0.089459 | 30.88 | <.0001* |
| Age (months) | 0.0023578 | 0.001092 | 2.16 | 0.0333* |

**Bivariate Fit of Ave PBR 20-25 By Weight (kg)**

**Linear Fit**

Ave PBR 20-25 = 2.6180859 + 0.0820744*Weight (kg)

**Summary of Fit**

| RSquare | 0.015835 |
| --- | --- |
| RSquare Adj | 0.005894 |
| Root Mean Square Error | 0.51419 |
| Mean of Response | 2.911782 |
| Observations (or Sum Wgts) | 101 |

**Analysis of Variance**

| **Source** | **DF** | **Sum of Squares** | **Mean Square** | **F Ratio** |
| --- | --- | --- | --- | --- |
| Model | 1 | 0.421155 | 0.421155 | 1.5929 |
| Error | 99 | 26.174724 | 0.264391 | **Prob > F** |
| C. Total | 100 | 26.595879 |  | 0.2099 |

**Parameter Estimates**

| **Term** | **Estimate** | **Std Error** | **t Ratio** | **Prob>\|t\|** |
| --- | --- | --- | --- | --- |
| Intercept | 2.6180859 | 0.238261 | 10.99 | <.0001* |
| Weight (kg) | 0.0820744 | 0.065029 | 1.26 | 0.2099 |

**Bivariate Fit of Ave PBR 20-25 By HR (b/min)**

**Linear Fit**

Ave PBR 20-25 = 2.6218882 + 0.0024143*HR (b/min)

**Summary of Fit**

| RSquare | 0.00823 |
| --- | --- |
| RSquare Adj | -0.00179 |
| Root Mean Square Error | 0.516173 |
| Mean of Response | 2.911782 |
| Observations (or Sum Wgts) | 101 |

**Analysis of Variance**

| **Source** | **DF** | **Sum of Squares** | **Mean Square** | **F Ratio** |
| --- | --- | --- | --- | --- |
| Model | 1 | 0.218893 | 0.218893 | 0.8216 |
| Error | 99 | 26.376986 | 0.266434 | **Prob > F** |
| C. Total | 100 | 26.595879 |  | 0.3669 |

**Parameter Estimates**

| **Term** | **Estimate** | **Std Error** | **t Ratio** | **Prob>\|t\|** |
| --- | --- | --- | --- | --- |
| Intercept | 2.6218882 | 0.323927 | 8.09 | <.0001* |
| HR (b/min) | 0.0024143 | 0.002664 | 0.91 | 0.3669 |

**Bivariate Fit of Ave PBR 20-25 By SpO2 (%)**

**Linear Fit**

Ave PBR 20-25 = -0.831825 + 0.0392265*SpO2 (%)

**Summary of Fit**

| RSquare | 0.015917 |
| --- | --- |
| RSquare Adj | 0.005976 |
| Root Mean Square Error | 0.514169 |
| Mean of Response | 2.911782 |
| Observations (or Sum Wgts) | 101 |

**Analysis of Variance**

| **Source** | **DF** | **Sum of Squares** | **Mean Square** | **F Ratio** |
| --- | --- | --- | --- | --- |
| Model | 1 | 0.423316 | 0.423316 | 1.6012 |
| Error | 99 | 26.172563 | 0.264369 | **Prob > F** |
| C. Total | 100 | 26.595879 |  | 0.2087 |

**Parameter Estimates**

| **Term** | **Estimate** | **Std Error** | **t Ratio** | **Prob>\|t\|** |
| --- | --- | --- | --- | --- |
| Intercept | -0.831825 | 2.958886 | -0.28 | 0.7792 |
| SpO2 (%) | 0.0392265 | 0.030999 | 1.27 | 0.2087 |

**Bivariate Fit of Ave PBR 20-25 By SAP (mmHg)**

**Linear Fit**

Ave PBR 20-25 = 3.2715821 - 0.0031049*SAP (mmHg)

**Summary of Fit**

| RSquare | 0.008506 |
| --- | --- |
| RSquare Adj | -0.00151 |
| Root Mean Square Error | 0.516101 |
| Mean of Response | 2.911782 |
| Observations (or Sum Wgts) | 101 |

**Analysis of Variance**

| **Source** | **DF** | **Sum of Squares** | **Mean Square** | **F Ratio** |
| --- | --- | --- | --- | --- |
| Model | 1 | 0.226237 | 0.226237 | 0.8494 |
| Error | 99 | 26.369642 | 0.266360 | **Prob > F** |
| C. Total | 100 | 26.595879 |  | 0.3590 |

**Parameter Estimates**

| **Term** | **Estimate** | **Std Error** | **t Ratio** | **Prob>\|t\|** |
| --- | --- | --- | --- | --- |
| Intercept | 3.2715821 | 0.393767 | 8.31 | <.0001* |
| SAP (mmHg) | -0.003105 | 0.003369 | -0.92 | 0.3590 |

**Bivariate Fit of Ave PBR 20-25 By DAP (mmHg)**

**Linear Fit**

Ave PBR 20-25 = 2.8472699 + 0.0010282*DAP (mmHg)

**Summary of Fit**

| RSquare | 0.000842 |
| --- | --- |
| RSquare Adj | -0.00925 |
| Root Mean Square Error | 0.518092 |
| Mean of Response | 2.911782 |
| Observations (or Sum Wgts) | 101 |

**Analysis of Variance**

| **Source** | **DF** | **Sum of Squares** | **Mean Square** | **F Ratio** |
| --- | --- | --- | --- | --- |
| Model | 1 | 0.022396 | 0.022396 | 0.0834 |
| Error | 99 | 26.573484 | 0.268419 | **Prob > F** |
| C. Total | 100 | 26.595879 |  | 0.7733 |

**Parameter Estimates**

| **Term** | **Estimate** | **Std Error** | **t Ratio** | **Prob>\|t\|** |
| --- | --- | --- | --- | --- |
| Intercept | 2.8472699 | 0.229213 | 12.42 | <.0001* |
| DAP (mmHg) | 0.0010282 | 0.003559 | 0.29 | 0.7733 |

**Bivariate Fit of Ave PBR 20-25 By MAP (mmHg)**

**Linear Fit**

Ave PBR 20-25 = 2.9762745 - 0.0008001*MAP (mmHg)

**Summary of Fit**

| RSquare | 0.000453 |
| --- | --- |
| RSquare Adj | -0.00964 |
| Root Mean Square Error | 0.518193 |
| Mean of Response | 2.911782 |
| Observations (or Sum Wgts) | 101 |

**Analysis of Variance**

| **Source** | **DF** | **Sum of Squares** | **Mean Square** | **F Ratio** |
| --- | --- | --- | --- | --- |
| Model | 1 | 0.012039 | 0.012039 | 0.0448 |
| Error | 99 | 26.583840 | 0.268524 | **Prob > F** |
| C. Total | 100 | 26.595879 |  | 0.8327 |

**Parameter Estimates**

| **Term** | **Estimate** | **Std Error** | **t Ratio** | **Prob>\|t\|** |
| --- | --- | --- | --- | --- |
| Intercept | 2.9762745 | 0.308911 | 9.63 | <.0001* |
| MAP (mmHg) | -0.0008 | 0.003779 | -0.21 | 0.8327 |

**Bivariate Fit of Ave PBR 20-25 By RR (bpm)**

**Linear Fit**

Ave PBR 20-25 = 2.8501156 + 0.003274*RR (bpm)

**Summary of Fit**

| RSquare | 0.000956 |
| --- | --- |
| RSquare Adj | -0.00914 |
| Root Mean Square Error | 0.518062 |
| Mean of Response | 2.911782 |
| Observations (or Sum Wgts) | 101 |

**Analysis of Variance**

| **Source** | **DF** | **Sum of Squares** | **Mean Square** | **F Ratio** |
| --- | --- | --- | --- | --- |
| Model | 1 | 0.025417 | 0.025417 | 0.0947 |
| Error | 99 | 26.570463 | 0.268389 | **Prob > F** |
| C. Total | 100 | 26.595879 |  | 0.7589 |

**Parameter Estimates**

| **Term** | **Estimate** | **Std Error** | **t Ratio** | **Prob>\|t\|** |
| --- | --- | --- | --- | --- |
| Intercept | 2.8501156 | 0.206913 | 13.77 | <.0001* |
| RR (bpm) | 0.003274 | 0.010639 | 0.31 | 0.7589 |

**Bivariate Fit of Ave PBR 20-25 By Temp ©**

**Linear Fit**

Ave PBR 20-25 = 0.74744 + 0.057132*Temp ©

**Summary of Fit**

| RSquare | 0.004518 |
| --- | --- |
| RSquare Adj | -0.00554 |
| Root Mean Square Error | 0.517138 |
| Mean of Response | 2.911782 |
| Observations (or Sum Wgts) | 101 |

**Analysis of Variance**

| **Source** | **DF** | **Sum of Squares** | **Mean Square** | **F Ratio** |
| --- | --- | --- | --- | --- |
| Model | 1 | 0.120169 | 0.120169 | 0.4493 |
| Error | 99 | 26.475710 | 0.267431 | **Prob > F** |
| C. Total | 100 | 26.595879 |  | 0.5042 |

**Parameter Estimates**

| **Term** | **Estimate** | **Std Error** | **t Ratio** | **Prob>\|t\|** |
| --- | --- | --- | --- | --- |
| Intercept | 0.74744 | 3.229166 | 0.23 | 0.8174 |
| Temp © | 0.057132 | 0.085229 | 0.67 | 0.5042 |

**Oneway Analysis of Ave PBR 20-25 By Sex**

**Oneway Anova**

**Summary of Fit**

| Rsquare | 0.037559 |
| --- | --- |
| Adj Rsquare | 0.007166 |
| Root Mean Square Error | 0.508258 |
| Mean of Response | 2.922525 |
| Observations (or Sum Wgts) | 99 |

**Analysis of Variance**

| **Source** | **DF** | **Sum of Squares** | **Mean Square** | **F Ratio** | **Prob > F** |
| --- | --- | --- | --- | --- | --- |
| Sex | 3 | 0.957704 | 0.319235 | 1.2358 | 0.3011 |
| Error | 95 | 24.540965 | 0.258326 |  |  |
| C. Total | 98 | 25.498669 |  |  |  |

**Means and Std Deviations**

| **Level** | **Number** | **Mean** | **Std Dev** | **Std Err Mean** | **Lower 95%** | **Upper 95%** |
| --- | --- | --- | --- | --- | --- | --- |
| 0 | 35 | 3.0362857 | 0.3780918 | 0.0639092 | 2.9064066 | 3.1661648 |
| 1 | 10 | 2.91 | 0.8037413 | 0.2541653 | 2.3350381 | 3.4849619 |
| 2 | 17 | 2.7552941 | 0.6502991 | 0.1577207 | 2.4209412 | 3.0896471 |
| 3 | 37 | 2.8951351 | 0.4441072 | 0.0730108 | 2.7470624 | 3.0432079 |

**Means Comparisons**

**Comparisons for all pairs using Tukey-Kramer HSD**

**Connecting Letters Report**

| **Level** |  | **Mean** |
| --- | --- | --- |
| 0 | A | 3.0362857 |
| 1 | A | 2.9100000 |
| 3 | A | 2.8951351 |
| 2 | A | 2.7552941 |

Levels not connected by same letter are significantly different.

Missing Rows2**Oneway Analysis of Ave PBR 20-25 By Pre-med protocol**

**Oneway Anova**

**Summary of Fit**

| Rsquare | 0.00194 |
| --- | --- |
| Adj Rsquare | -0.00814 |
| Root Mean Square Error | 0.517807 |
| Mean of Response | 2.911782 |
| Observations (or Sum Wgts) | 101 |

**Analysis of Variance**

| **Source** | **DF** | **Sum of Squares** | **Mean Square** | **F Ratio** | **Prob > F** |
| --- | --- | --- | --- | --- | --- |
| Pre-med protocol | 1 | 0.051592 | 0.051592 | 0.1924 | 0.6619 |
| Error | 99 | 26.544287 | 0.268124 |  |  |
| C. Total | 100 | 26.595879 |  |  |  |

**Means and Std Deviations**

| **Level** | **Number** | **Mean** | **Std Dev** | **Std Err Mean** | **Lower 95%** | **Upper 95%** |
| --- | --- | --- | --- | --- | --- | --- |
| 1 | 12 | 2.9733333 | 0.7144525 | 0.2062447 | 2.5193918 | 3.4272748 |
| 2 | 89 | 2.9034831 | 0.4876826 | 0.0516943 | 2.8007517 | 3.0062146 |

**Means Comparisons**

**Comparisons for all pairs using Tukey-Kramer HSD**

**Bivariate Fit of Ave Median P50 By Age (months)**

**Linear Fit**

Ave Median P50 = 7.2260562 - 0.0028307*Age (months)

**Summary of Fit**

| RSquare | 0.036911 |
| --- | --- |
| RSquare Adj | 0.026982 |
| Root Mean Square Error | 0.673336 |
| Mean of Response | 7.034343 |
| Observations (or Sum Wgts) | 99 |

**Analysis of Variance**

| **Source** | **DF** | **Sum of Squares** | **Mean Square** | **F Ratio** |
| --- | --- | --- | --- | --- |
| Model | 1 | 1.685486 | 1.68549 | 3.7176 |
| Error | 97 | 43.978052 | 0.45338 | **Prob > F** |
| C. Total | 98 | 45.663538 |  | 0.0568 |

**Parameter Estimates**

| **Term** | **Estimate** | **Std Error** | **t Ratio** | **Prob>\|t\|** |
| --- | --- | --- | --- | --- |
| Intercept | 7.2260562 | 0.120275 | 60.08 | <.0001* |
| Age (months) | -0.002831 | 0.001468 | -1.93 | 0.0568 |

**Bivariate Fit of Ave Median P50 By Weight (kg)**

**Linear Fit**

Ave Median P50 = 7.8423016 - 0.2287988*Weight (kg)

**Summary of Fit**

| RSquare | 0.070749 |
| --- | --- |
| RSquare Adj | 0.061363 |
| Root Mean Square Error | 0.658953 |
| Mean of Response | 7.023564 |
| Observations (or Sum Wgts) | 101 |

**Analysis of Variance**

| **Source** | **DF** | **Sum of Squares** | **Mean Square** | **F Ratio** |
| --- | --- | --- | --- | --- |
| Model | 1 | 3.272913 | 3.27291 | 7.5375 |
| Error | 99 | 42.987710 | 0.43422 | **Prob > F** |
| C. Total | 100 | 46.260622 |  | 0.0072* |

**Parameter Estimates**

| **Term** | **Estimate** | **Std Error** | **t Ratio** | **Prob>\|t\|** |
| --- | --- | --- | --- | --- |
| Intercept | 7.8423016 | 0.30534 | 25.68 | <.0001* |
| Weight (kg) | -0.228799 | 0.083338 | -2.75 | 0.0072* |

**Bivariate Fit of Ave Median P50 By HR (b/min)**

**Linear Fit**

Ave Median P50 = 7.2662474 - 0.0020211*HR (b/min)

**Summary of Fit**

| RSquare | 0.003316 |
| --- | --- |
| RSquare Adj | -0.00675 |
| Root Mean Square Error | 0.682444 |
| Mean of Response | 7.023564 |
| Observations (or Sum Wgts) | 101 |

**Analysis of Variance**

| **Source** | **DF** | **Sum of Squares** | **Mean Square** | **F Ratio** |
| --- | --- | --- | --- | --- |
| Model | 1 | 0.153402 | 0.153402 | 0.3294 |
| Error | 99 | 46.107220 | 0.465729 | **Prob > F** |
| C. Total | 100 | 46.260622 |  | 0.5673 |

**Parameter Estimates**

| **Term** | **Estimate** | **Std Error** | **t Ratio** | **Prob>\|t\|** |
| --- | --- | --- | --- | --- |
| Intercept | 7.2662474 | 0.428271 | 16.97 | <.0001* |
| HR (b/min) | -0.002021 | 0.003522 | -0.57 | 0.5673 |

**Bivariate Fit of Ave Median P50 By SpO2 (%)**

**Linear Fit**

Ave Median P50 = 13.421047 - 0.0670345*SpO2 (%)

**Summary of Fit**

| RSquare | 0.026723 |
| --- | --- |
| RSquare Adj | 0.016892 |
| Root Mean Square Error | 0.674382 |
| Mean of Response | 7.023564 |
| Observations (or Sum Wgts) | 101 |

**Analysis of Variance**

| **Source** | **DF** | **Sum of Squares** | **Mean Square** | **F Ratio** |
| --- | --- | --- | --- | --- |
| Model | 1 | 1.236239 | 1.23624 | 2.7183 |
| Error | 99 | 45.024383 | 0.45479 | **Prob > F** |
| C. Total | 100 | 46.260622 |  | 0.1024 |

**Parameter Estimates**

| **Term** | **Estimate** | **Std Error** | **t Ratio** | **Prob>\|t\|** |
| --- | --- | --- | --- | --- |
| Intercept | 13.421047 | 3.88087 | 3.46 | 0.0008* |
| SpO2 (%) | -0.067035 | 0.040659 | -1.65 | 0.1024 |

**Bivariate Fit of Ave Median P50 By SAP (mmHg)**

**Linear Fit**

Ave Median P50 = 7.1487455 - 0.0010803*SAP (mmHg)

**Summary of Fit**

| RSquare | 0.000592 |
| --- | --- |
| RSquare Adj | -0.0095 |
| Root Mean Square Error | 0.683376 |
| Mean of Response | 7.023564 |
| Observations (or Sum Wgts) | 101 |

**Analysis of Variance**

| **Source** | **DF** | **Sum of Squares** | **Mean Square** | **F Ratio** |
| --- | --- | --- | --- | --- |
| Model | 1 | 0.027385 | 0.027385 | 0.0586 |
| Error | 99 | 46.233237 | 0.467002 | **Prob > F** |
| C. Total | 100 | 46.260622 |  | 0.8092 |

**Parameter Estimates**

| **Term** | **Estimate** | **Std Error** | **t Ratio** | **Prob>\|t\|** |
| --- | --- | --- | --- | --- |
| Intercept | 7.1487455 | 0.521392 | 13.71 | <.0001* |
| SAP (mmHg) | -0.00108 | 0.004461 | -0.24 | 0.8092 |

**Bivariate Fit of Ave Median P50 By DAP (mmHg)**

**Linear Fit**

Ave Median P50 = 7.5843423 - 0.0089373*DAP (mmHg)

**Summary of Fit**

| RSquare | 0.03658 |
| --- | --- |
| RSquare Adj | 0.026849 |
| Root Mean Square Error | 0.670959 |
| Mean of Response | 7.023564 |
| Observations (or Sum Wgts) | 101 |

**Analysis of Variance**

| **Source** | **DF** | **Sum of Squares** | **Mean Square** | **F Ratio** |
| --- | --- | --- | --- | --- |
| Model | 1 | 1.692232 | 1.69223 | 3.7590 |
| Error | 99 | 44.568390 | 0.45019 | **Prob > F** |
| C. Total | 100 | 46.260622 |  | 0.0554 |

**Parameter Estimates**

| **Term** | **Estimate** | **Std Error** | **t Ratio** | **Prob>\|t\|** |
| --- | --- | --- | --- | --- |
| Intercept | 7.5843423 | 0.296844 | 25.55 | <.0001* |
| DAP (mmHg) | -0.008937 | 0.00461 | -1.94 | 0.0554 |

**Bivariate Fit of Ave Median P50 By MAP (mmHg)**

**Linear Fit**

Ave Median P50 = 7.5811621 - 0.0069175*MAP (mmHg)

**Summary of Fit**

| RSquare | 0.019454 |
| --- | --- |
| RSquare Adj | 0.00955 |
| Root Mean Square Error | 0.676896 |
| Mean of Response | 7.023564 |
| Observations (or Sum Wgts) | 101 |

**Analysis of Variance**

| **Source** | **DF** | **Sum of Squares** | **Mean Square** | **F Ratio** |
| --- | --- | --- | --- | --- |
| Model | 1 | 0.899971 | 0.899971 | 1.9642 |
| Error | 99 | 45.360651 | 0.458188 | **Prob > F** |
| C. Total | 100 | 46.260622 |  | 0.1642 |

**Parameter Estimates**

| **Term** | **Estimate** | **Std Error** | **t Ratio** | **Prob>\|t\|** |
| --- | --- | --- | --- | --- |
| Intercept | 7.5811621 | 0.40352 | 18.79 | <.0001* |
| MAP (mmHg) | -0.006918 | 0.004936 | -1.40 | 0.1642 |

**Bivariate Fit of Ave Median P50 By RR (bpm)**

**Linear Fit**

Ave Median P50 = 6.8217879 + 0.0107129*RR (bpm)

**Summary of Fit**

| RSquare | 0.005882 |
| --- | --- |
| RSquare Adj | -0.00416 |
| Root Mean Square Error | 0.681565 |
| Mean of Response | 7.023564 |
| Observations (or Sum Wgts) | 101 |

**Analysis of Variance**

| **Source** | **DF** | **Sum of Squares** | **Mean Square** | **F Ratio** |
| --- | --- | --- | --- | --- |
| Model | 1 | 0.272118 | 0.272118 | 0.5858 |
| Error | 99 | 45.988504 | 0.464530 | **Prob > F** |
| C. Total | 100 | 46.260622 |  | 0.4459 |

**Parameter Estimates**

| **Term** | **Estimate** | **Std Error** | **t Ratio** | **Prob>\|t\|** |
| --- | --- | --- | --- | --- |
| Intercept | 6.8217879 | 0.272216 | 25.06 | <.0001* |
| RR (bpm) | 0.0107129 | 0.013997 | 0.77 | 0.4459 |

**Bivariate Fit of Ave Median P50 By Temp ©**

**Linear Fit**

Ave Median P50 = 6.0956961 + 0.0244929*Temp ©

**Summary of Fit**

| RSquare | 0.000477 |
| --- | --- |
| RSquare Adj | -0.00962 |
| Root Mean Square Error | 0.683415 |
| Mean of Response | 7.023564 |
| Observations (or Sum Wgts) | 101 |

**Analysis of Variance**

| **Source** | **DF** | **Sum of Squares** | **Mean Square** | **F Ratio** |
| --- | --- | --- | --- | --- |
| Model | 1 | 0.022086 | 0.022086 | 0.0473 |
| Error | 99 | 46.238537 | 0.467056 | **Prob > F** |
| C. Total | 100 | 46.260622 |  | 0.8283 |

**Parameter Estimates**

| **Term** | **Estimate** | **Std Error** | **t Ratio** | **Prob>\|t\|** |
| --- | --- | --- | --- | --- |
| Intercept | 6.0956961 | 4.267451 | 1.43 | 0.1563 |
| Temp © | 0.0244929 | 0.112633 | 0.22 | 0.8283 |

**Oneway Analysis of Ave Median P50 By Sex**

**Oneway Anova**

**Summary of Fit**

| Rsquare | 0.08739 |
| --- | --- |
| Adj Rsquare | 0.058571 |
| Root Mean Square Error | 0.662316 |
| Mean of Response | 7.034343 |
| Observations (or Sum Wgts) | 99 |

**Analysis of Variance**

| **Source** | **DF** | **Sum of Squares** | **Mean Square** | **F Ratio** | **Prob > F** |
| --- | --- | --- | --- | --- | --- |
| Sex | 3 | 3.990554 | 1.33018 | 3.0324 | 0.0331* |
| Error | 95 | 41.672983 | 0.43866 |  |  |
| C. Total | 98 | 45.663538 |  |  |  |

**Means and Std Deviations**

| **Level** | **Number** | **Mean** | **Std Dev** | **Std Err Mean** | **Lower 95%** | **Upper 95%** |
| --- | --- | --- | --- | --- | --- | --- |
| 0 | 35 | 6.9685714 | 0.6700528 | 0.1132596 | 6.7384002 | 7.1987426 |
| 1 | 10 | 6.9771667 | 0.6624944 | 0.2094991 | 6.5032467 | 7.4510866 |
| 2 | 17 | 6.7038235 | 0.4624639 | 0.112164 | 6.4660465 | 6.9416005 |
| 3 | 37 | 7.2638739 | 0.7271696 | 0.1195459 | 7.0214235 | 7.5063243 |

**Means Comparisons**

**Comparisons for all pairs using Tukey-Kramer HSD**

**Connecting Letters Report**

| **Level** |  |  | **Mean** |
| --- | --- | --- | --- |
| 3 | A |  | 7.2638739 |
| 1 | A | B | 6.9771667 |
| 0 | A | B | 6.9685714 |
| 2 |  | B | 6.7038235 |

Levels not connected by same letter are significantly different.

Missing Rows2**Oneway Analysis of Ave Median P50 By Pre-med protocol**

**Oneway Anova**

**Summary of Fit**

| Rsquare | 0.006767 |
| --- | --- |
| Adj Rsquare | -0.00327 |
| Root Mean Square Error | 0.681261 |
| Mean of Response | 7.023564 |
| Observations (or Sum Wgts) | 101 |

**Analysis of Variance**

| **Source** | **DF** | **Sum of Squares** | **Mean Square** | **F Ratio** | **Prob > F** |
| --- | --- | --- | --- | --- | --- |
| Pre-med protocol | 1 | 0.313058 | 0.313058 | 0.6745 | 0.4135 |
| Error | 99 | 45.947564 | 0.464117 |  |  |
| C. Total | 100 | 46.260622 |  |  |  |

**Means and Std Deviations**

| **Level** | **Number** | **Mean** | **Std Dev** | **Std Err Mean** | **Lower 95%** | **Upper 95%** |
| --- | --- | --- | --- | --- | --- | --- |
| 1 | 12 | 6.8719444 | 0.4095093 | 0.1182152 | 6.6117546 | 7.1321343 |
| 2 | 89 | 7.0440075 | 0.707933 | 0.0750408 | 6.8948798 | 7.1931352 |

**Means Comparisons**

**Comparisons for all pairs using Tukey-Kramer HSD**
