# Supplementary material for: Rapid Patient-Side Evaluation of Endothelial Glycocalyx Thickness in Healthy Sedated Cats Using GlycoCheck® Software
Source: Front Vet Sci. 2022 Jan 3;8:727063. doi: 10.3389/fvets.2021.727063 (PMC8761653; doi:10.3389/fvets.2021.727063)
Supplement: Supplementary file 4 [file Data_Sheet_2.docx]

**Oneway Analysis of Ave vessel density By Pre-med protocol**

**Oneway Anova**

**Summary of Fit**

| Rsquare | 0.000405 |
| --- | --- |
| Adj Rsquare | -0.00969 |
| Root Mean Square Error | 65.37579 |
| Mean of Response | 171.2688 |
| Observations (or Sum Wgts) | 101 |

**Analysis of Variance**

| **Source** | **DF** | **Sum of Squares** | **Mean Square** | **F Ratio** | **Prob > F** |
| --- | --- | --- | --- | --- | --- |
| Pre-med protocol | 1 | 171.26 | 171.26 | 0.0401 | 0.8418 |
| Error | 99 | 423125.39 | 4273.99 |  |  |
| C. Total | 100 | 423296.65 |  |  |  |

**Means and Std Deviations**

| **Level** | **Number** | **Mean** | **Std Dev** | **Std Err Mean** | **Lower 95%** | **Upper 95%** |
| --- | --- | --- | --- | --- | --- | --- |
| 1 | 12 | 167.7225 | 76.342994 | 22.038324 | 119.21648 | 216.22852 |
| 2 | 89 | 171.74697 | 63.872619 | 6.7704841 | 158.29205 | 185.20188 |

**Oneway Analysis of Ave RBC filling % By Pre-med protocol**

**Oneway Anova**

**Summary of Fit**

| Rsquare | 0.020913 |
| --- | --- |
| Adj Rsquare | 0.011024 |
| Root Mean Square Error | 5.889513 |
| Mean of Response | 73.07416 |
| Observations (or Sum Wgts) | 101 |

**Analysis of Variance**

| **Source** | **DF** | **Sum of Squares** | **Mean Square** | **F Ratio** | **Prob > F** |
| --- | --- | --- | --- | --- | --- |
| Pre-med protocol | 1 | 73.3495 | 73.3495 | 2.1147 | 0.1491 |
| Error | 99 | 3433.9495 | 34.6864 |  |  |
| C. Total | 100 | 3507.2991 |  |  |  |

**Means and Std Deviations**

| **Level** | **Number** | **Mean** | **Std Dev** | **Std Err Mean** | **Lower 95%** | **Upper 95%** |
| --- | --- | --- | --- | --- | --- | --- |
| 1 | 12 | 70.753333 | 6.7298593 | 1.942743 | 66.477385 | 75.029282 |
| 2 | 89 | 73.387079 | 5.775879 | 0.6122419 | 72.170376 | 74.603781 |

**Means Comparisons**

**Comparisons for all pairs using Tukey-Kramer HSD**

**Oneway Analysis of Ave PBR 5-25 By Pre-med protocol**

**Oneway Anova**

**Summary of Fit**

| Rsquare | 0.017532 |
| --- | --- |
| Adj Rsquare | 0.007608 |
| Root Mean Square Error | 0.243831 |
| Mean of Response | 2.372178 |
| Observations (or Sum Wgts) | 101 |

**Analysis of Variance**

| **Source** | **DF** | **Sum of Squares** | **Mean Square** | **F Ratio** | **Prob > F** |
| --- | --- | --- | --- | --- | --- |
| Pre-med protocol | 1 | 0.1050309 | 0.105031 | 1.7666 | 0.1869 |
| Error | 99 | 5.8858899 | 0.059453 |  |  |
| C. Total | 100 | 5.9909208 |  |  |  |

**Means and Std Deviations**

| **Level** | **Number** | **Mean** | **Std Dev** | **Std Err Mean** | **Lower 95%** | **Upper 95%** |
| --- | --- | --- | --- | --- | --- | --- |
| 1 | 12 | 2.46 | 0.3279412 | 0.0946685 | 2.2516361 | 2.6683639 |
| 2 | 89 | 2.3603371 | 0.2311751 | 0.0245045 | 2.3116395 | 2.4090346 |

**Oneway Analysis of Ave PBR 5-9 By Pre-med protocol**

**Oneway Anova**

**Summary of Fit**

| Rsquare | 0.001706 |
| --- | --- |
| Adj Rsquare | -0.00838 |
| Root Mean Square Error | 0.147451 |
| Mean of Response | 1.346436 |
| Observations (or Sum Wgts) | 101 |

**Analysis of Variance**

| **Source** | **DF** | **Sum of Squares** | **Mean Square** | **F Ratio** | **Prob > F** |
| --- | --- | --- | --- | --- | --- |
| Pre-med protocol | 1 | 0.0036786 | 0.003679 | 0.1692 | 0.6817 |
| Error | 99 | 2.1524382 | 0.021742 |  |  |
| C. Total | 100 | 2.1561168 |  |  |  |

**Means and Std Deviations**

| **Level** | **Number** | **Mean** | **Std Dev** | **Std Err Mean** | **Lower 95%** | **Upper 95%** |
| --- | --- | --- | --- | --- | --- | --- |
| 1 | 12 | 1.33 | 0.1561468 | 0.0450757 | 1.2307891 | 1.4292109 |
| 2 | 89 | 1.3486517 | 0.1463277 | 0.0155107 | 1.3178274 | 1.379476 |

**Oneway Analysis of Ave PBR 10-19 By Pre-med protocol**

**Oneway Anova**

**Summary of Fit**

| Rsquare | 0.021475 |
| --- | --- |
| Adj Rsquare | 0.01159 |
| Root Mean Square Error | 0.293841 |
| Mean of Response | 2.693465 |
| Observations (or Sum Wgts) | 101 |

**Analysis of Variance**

| **Source** | **DF** | **Sum of Squares** | **Mean Square** | **F Ratio** | **Prob > F** |
| --- | --- | --- | --- | --- | --- |
| Pre-med protocol | 1 | 0.1875910 | 0.187591 | 2.1726 | 0.1437 |
| Error | 99 | 8.5478962 | 0.086342 |  |  |
| C. Total | 100 | 8.7354871 |  |  |  |

**Means and Std Deviations**

| **Level** | **Number** | **Mean** | **Std Dev** | **Std Err Mean** | **Lower 95%** | **Upper 95%** |
| --- | --- | --- | --- | --- | --- | --- |
| 1 | 12 | 2.8108333 | 0.3937802 | 0.1136746 | 2.5606373 | 3.0610294 |
| 2 | 89 | 2.6776404 | 0.278841 | 0.0295571 | 2.6189019 | 2.736379 |

**Means Comparisons**

**Comparisons for all pairs using Tukey-Kramer HSD**

**Oneway Analysis of Ave PBR 20-25 By Pre-med protocol**

**Oneway Anova**

**Summary of Fit**

| Rsquare | 0.00194 |
| --- | --- |
| Adj Rsquare | -0.00814 |
| Root Mean Square Error | 0.517807 |
| Mean of Response | 2.911782 |
| Observations (or Sum Wgts) | 101 |

**Analysis of Variance**

| **Source** | **DF** | **Sum of Squares** | **Mean Square** | **F Ratio** | **Prob > F** |
| --- | --- | --- | --- | --- | --- |
| Pre-med protocol | 1 | 0.051592 | 0.051592 | 0.1924 | 0.6619 |
| Error | 99 | 26.544287 | 0.268124 |  |  |
| C. Total | 100 | 26.595879 |  |  |  |

**Means and Std Deviations**

| **Level** | **Number** | **Mean** | **Std Dev** | **Std Err Mean** | **Lower 95%** | **Upper 95%** |
| --- | --- | --- | --- | --- | --- | --- |
| 1 | 12 | 2.9733333 | 0.7144525 | 0.2062447 | 2.5193918 | 3.4272748 |
| 2 | 89 | 2.9034831 | 0.4876826 | 0.0516943 | 2.8007517 | 3.0062146 |

**Means Comparisons**

**Comparisons for all pairs using Tukey-Kramer HSD**

**Oneway Analysis of Ave Median P50 By Pre-med protocol**

**Oneway Anova**

**Summary of Fit**

| Rsquare | 0.006767 |
| --- | --- |
| Adj Rsquare | -0.00327 |
| Root Mean Square Error | 0.681261 |
| Mean of Response | 7.023564 |
| Observations (or Sum Wgts) | 101 |

**Analysis of Variance**

| **Source** | **DF** | **Sum of Squares** | **Mean Square** | **F Ratio** | **Prob > F** |
| --- | --- | --- | --- | --- | --- |
| Pre-med protocol | 1 | 0.313058 | 0.313058 | 0.6745 | 0.4135 |
| Error | 99 | 45.947564 | 0.464117 |  |  |
| C. Total | 100 | 46.260622 |  |  |  |

**Means and Std Deviations**

| **Level** | **Number** | **Mean** | **Std Dev** | **Std Err Mean** | **Lower 95%** | **Upper 95%** |
| --- | --- | --- | --- | --- | --- | --- |
| 1 | 12 | 6.8719444 | 0.4095093 | 0.1182152 | 6.6117546 | 7.1321343 |
| 2 | 89 | 7.0440075 | 0.707933 | 0.0750408 | 6.8948798 | 7.1931352 |

**Means Comparisons**

**Comparisons for all pairs using Tukey-Kramer HSD**
