# Supplementary material for: Rapid Patient-Side Evaluation of Endothelial Glycocalyx Thickness in Healthy Sedated Cats Using GlycoCheck® Software
Source: Front Vet Sci. 2022 Jan 3;8:727063. doi: 10.3389/fvets.2021.727063 (PMC8761653; doi:10.3389/fvets.2021.727063)
Supplement: Supplementary file 5 [file Data_Sheet_3.docx]

Ave vessel density

| **Coverage** | **BC Lower** | **BC Upper** |
| --- | --- | --- |
| 0.95 | 265.186 | 320.451 |
| 0.90 | 269.558 | 316.643 |
| 0.80 | 275.313 | 311.63 |
| 0.50 | 284.028 | 303.344 |

| Original Estimate | 293.1572 |
| --- | --- |

| **Coverage** | **BC Lower** | **BC Upper** |
| --- | --- | --- |
| 0.95 | 34.5801 | 62.6777 |
| 0.90 | 37.1746 | 60.1317 |
| 0.80 | 39.2955 | 57.6029 |
| 0.50 | 43.6767 | 53.1583 |

| Original Estimate | 49.38046 |
| --- | --- |

Ave RBC filling %

| **Coverage** | **BC Lower** | **BC Upper** |
| --- | --- | --- |
| 0.95 | 82.4026 | 86.1986 |
| 0.90 | 82.7221 | 85.9011 |
| 0.80 | 83.0693 | 85.5375 |
| 0.50 | 83.6271 | 84.9661 |

| Original Estimate | 84.16913 |
| --- | --- |

| **Coverage** | **BC Lower** | **BC Upper** |
| --- | --- | --- |
| 0.95 | 60.253 | 63.6012 |
| 0.90 | 60.4959 | 63.3162 |
| 0.80 | 60.8051 | 63.006 |
| 0.50 | 61.3281 | 62.476 |

| Original Estimate | 61.97919 |
| --- | --- |

Ave PBR 5-25

| **Coverage** | **BC Lower** | **BC Upper** |
| --- | --- | --- |
| 0.95 | 2.75697 | 2.91732 |
| 0.90 | 2.76971 | 2.90263 |
| 0.80 | 2.78379 | 2.88877 |
| 0.50 | 2.80776 | 2.86477 |

| Original Estimate | 2.830728 |
| --- | --- |
|  |  |

| **Coverage** | **BC Lower** | **BC Upper** |
| --- | --- | --- |
| 0.95 | 1.83824 | 1.98003 |
| 0.90 | 1.84795 | 1.96941 |
| 0.80 | 1.86178 | 1.95579 |
| 0.50 | 1.88435 | 1.93356 |

| Original Estimate | 1.913628 |
| --- | --- |

Ave PBR 5-9

| **Coverage** | **BC Lower** | **BC Upper** |
| --- | --- | --- |
| 0.95 | 1.58452 | 1.66533 |
| 0.90 | 1.59105 | 1.65883 |
| 0.80 | 1.59884 | 1.65068 |
| 0.50 | 1.6111 | 1.63813 |

| Original Estimate | 1.621526 |
| --- | --- |
|  |  |

| **Coverage** | **BC Lower** | **BC Upper** |
| --- | --- | --- |
| 0.95 | 1.01555 | 1.12398 |
| 0.90 | 1.02311 | 1.11592 |
| 0.80 | 1.03424 | 1.10583 |
| 0.50 | 1.05096 | 1.08863 |

| Original Estimate | 1.071345 |
| --- | --- |

Ave PBR 10-19

| **Coverage** | **BC Lower** | **BC Upper** |
| --- | --- | --- |
| 0.95 | 3.15235 | 3.35943 |
| 0.90 | 3.17003 | 3.3421 |
| 0.80 | 3.18813 | 3.32257 |
| 0.50 | 3.21894 | 3.29043 |

| Original Estimate | 3.247177 |
| --- | --- |
|  |  |

| **Coverage** | **BC Lower** | **BC Upper** |
| --- | --- | --- |
| 0.95 | 2.04789 | 2.22387 |
| 0.90 | 2.06181 | 2.20787 |
| 0.80 | 2.07814 | 2.19378 |
| 0.50 | 2.10492 | 2.16644 |

| Original Estimate | 2.139754 |
| --- | --- |

Ave PBR 20-25

| **Coverage** | **BC Lower** | **BC Upper** |
| --- | --- | --- |
| 0.95 | 3.69093 | 4.09097 |
| 0.90 | 3.7173 | 4.05819 |
| 0.80 | 3.75057 | 4.01788 |
| 0.50 | 3.80983 | 3.95166 |

| Original Estimate | 3.877938 |
| --- | --- |

| **Coverage** | **BC Lower** | **BC Upper** |
| --- | --- | --- |
| 0.95 | 1.76567 | 2.09066 |
| 0.90 | 1.79313 | 2.0685 |
| 0.80 | 1.82936 | 2.04328 |
| 0.50 | 1.88081 | 1.99289 |

| Original Estimate | 1.945627 |
| --- | --- |

Median P50

| **Coverage** | **BC Lower** | **BC Upper** |
| --- | --- | --- |
| 0.95 | 8.04696 | 8.63283 |
| 0.90 | 8.08684 | 8.57636 |
| 0.80 | 8.13531 | 8.508 |
| 0.50 | 8.21837 | 8.42093 |

| Original Estimate | 8.297788 |
| --- | --- |

| **Coverage** | **BC Lower** | **BC Upper** |
| --- | --- | --- |
| 0.95 | 5.5126 | 5.967 |
| 0.90 | 5.54766 | 5.92902 |
| 0.80 | 5.58554 | 5.88946 |
| 0.50 | 5.65911 | 5.81793 |

| Original Estimate | 5.749341 |
| --- | --- |
