# Supplementary material for: Rapid Patient-Side Evaluation of Endothelial Glycocalyx Thickness in Healthy Sedated Cats Using GlycoCheck® Software
Source: Front Vet Sci. 2022 Jan 3;8:727063. doi: 10.3389/fvets.2021.727063 (PMC8761653; doi:10.3389/fvets.2021.727063)
Supplement: Supplementary file 6 [file Data_Sheet_4.docx]

**Response Ave PBR 5-25 Label=Age (months)**

**Whole Model**

**Lack Of Fit**

| **Source** | **DF** | **Sum of Squares** | **Mean Square** | **F Ratio** |
| --- | --- | --- | --- | --- |
| Lack Of Fit | 30 | 1.6221397 | 0.054071 | 0.9125 |
| Pure Error | 67 | 3.9701170 | 0.059255 | **Prob > F** |
| Total Error | 97 | 5.5922567 |  | 0.5994 |
|  |  |  |  | **Max RSq** |
|  |  |  |  | 0.3081 |

**Power Details**

Test Data

**Power**

| **α** | **σ** | **δ** | **Number** | **Power** | **AdjPower** | **LowerCL** | **UpperCL** |
| --- | --- | --- | --- | --- | --- | --- | --- |
| 0.0500 | 0.240109 | 0.038399 | 99 | 0.3505 | 0.2257 | 0.0500 | 0.9430 |

**Least Significant Number**

| **α** | **σ** | **δ** | **Number(LSN)** |
| --- | --- | --- | --- |
| 0.0500 | 0.240109 | 0.038399 | 152.6414 |

**Least Significant Value**

| **α** | **σ** | **Number** | **LSV** |
| --- | --- | --- | --- |
| 0.0500 | 0.240109 | 99 | 0.001039 |

**Response Ave PBR 5-25 Label=HR (b/min)**

**Whole Model**

**Lack Of Fit**

| **Source** | **DF** | **Sum of Squares** | **Mean Square** | **F Ratio** |
| --- | --- | --- | --- | --- |
| Lack Of Fit | 72 | 4.4012659 | 0.061129 | 1.1026 |
| Pure Error | 27 | 1.4968917 | 0.055440 | **Prob > F** |
| Total Error | 99 | 5.8981576 |  | 0.3999 |
|  |  |  |  | **Max RSq** |
|  |  |  |  | 0.7501 |

**Data**

**Power Details**

Test Data

**Power**

| **α** | **σ** | **δ** | **Number** | **Power** | **AdjPower** | **LowerCL** | **UpperCL** |
| --- | --- | --- | --- | --- | --- | --- | --- |
| 0.0500 | 0.244085 | 0.030306 | 101 | 0.2352 | 0.1108 | 0.0500 | 0.8926 |

**Least Significant Number**

| **α** | **σ** | **δ** | **Number(LSN)** |
| --- | --- | --- | --- |
| 0.0500 | 0.244085 | 0.030306 | 251.6196 |

**Least Significant Value**

| **α** | **σ** | **Number** | **LSV** |
| --- | --- | --- | --- |
| 0.0500 | 0.244085 | 101 | 0.002499 |

**Response Ave PBR 5-25 Label=RR (bpm)**

**Whole Model**

**Lack Of Fit**

| **Source** | **DF** | **Sum of Squares** | **Mean Square** | **F Ratio** |
| --- | --- | --- | --- | --- |
| Lack Of Fit | 25 | 1.9447293 | 0.077789 | 1.4267 |
| Pure Error | 74 | 4.0346644 | 0.054522 | **Prob > F** |
| Total Error | 99 | 5.9793937 |  | 0.1221 |
|  |  |  |  | **Max RSq** |
|  |  |  |  | 0.3265 |

**Data**

**Power Details**

Test Data

**Power**

| **α** | **σ** | **δ** | **Number** | **Power** | **AdjPower** | **LowerCL** | **UpperCL** |
| --- | --- | --- | --- | --- | --- | --- | --- |
| 0.0500 | 0.24576 | 0.010683 | 101 | 0.0717 | 0.0500 | 0.0500 | 0.6692 |

**Least Significant Number**

| **α** | **σ** | **δ** | **Number(LSN)** |
| --- | --- | --- | --- |
| 0.0500 | 0.24576 | 0.010683 | 2035.341 |

**Least Significant Value**

| **α** | **σ** | **Number** | **LSV** |
| --- | --- | --- | --- |
| 0.0500 | 0.24576 | 101 | 0.010014 |

**Response Ave PBR 5-25 Label=SpO2 (%)**

**Whole Model**

**Lack Of Fit**

| **Source** | **DF** | **Sum of Squares** | **Mean Square** | **F Ratio** |
| --- | --- | --- | --- | --- |
| Lack Of Fit | 13 | 0.9610635 | 0.073928 | 1.2826 |
| Pure Error | 86 | 4.9571473 | 0.057641 | **Prob > F** |
| Total Error | 99 | 5.9182109 |  | 0.2392 |
|  |  |  |  | **Max RSq** |
|  |  |  |  | 0.1726 |

**Data**

**Power Details**

Test Data

**Power**

| **α** | **σ** | **δ** | **Number** | **Power** | **AdjPower** | **LowerCL** | **UpperCL** |
| --- | --- | --- | --- | --- | --- | --- | --- |
| 0.0500 | 0.244499 | 0.026831 | 101 | 0.1939 | 0.0718 | 0.0500 | 0.8637 |

**Least Significant Number**

| **α** | **σ** | **δ** | **Number(LSN)** |
| --- | --- | --- | --- |
| 0.0500 | 0.244499 | 0.026831 | 321.4227 |

**Least Significant Value**

| **α** | **σ** | **Number** | **LSV** |
| --- | --- | --- | --- |
| 0.0500 | 0.244499 | 101 | 0.029249 |

**Response Ave PBR 5-25 Label=Temp ©**

**Whole Model**

**Lack Of Fit**

| **Source** | **DF** | **Sum of Squares** | **Mean Square** | **F Ratio** |
| --- | --- | --- | --- | --- |
| Lack Of Fit | 46 | 2.7163201 | 0.059050 | 0.9988 |
| Pure Error | 53 | 3.1334167 | 0.059121 | **Prob > F** |
| Total Error | 99 | 5.8497368 |  | 0.4990 |
|  |  |  |  | **Max RSq** |
|  |  |  |  | 0.4770 |

**Data**

**Power Details**

Test Data

**Power**

| **α** | **σ** | **δ** | **Number** | **Power** | **AdjPower** | **LowerCL** | **UpperCL** |
| --- | --- | --- | --- | --- | --- | --- | --- |
| 0.0500 | 0.243081 | 0.037388 | 101 | 0.3341 | 0.2090 | 0.0500 | 0.9377 |

**Least Significant Number**

| **α** | **σ** | **δ** | **Number(LSN)** |
| --- | --- | --- | --- |
| 0.0500 | 0.243081 | 0.037388 | 164.8213 |

**Least Significant Value**

| **α** | **σ** | **Number** | **LSV** |
| --- | --- | --- | --- |
| 0.0500 | 0.243081 | 101 | 0.079492 |

**Response Ave PBR 5-25 Label=Weight (kg)**

**Whole Model**

**Lack Of Fit**

| **Source** | **DF** | **Sum of Squares** | **Mean Square** | **F Ratio** |
| --- | --- | --- | --- | --- |
| Lack Of Fit | 95 | 5.8101053 | 0.061159 | 1.8297 |
| Pure Error | 4 | 0.1337000 | 0.033425 | **Prob > F** |
| Total Error | 99 | 5.9438053 |  | 0.2980 |
|  |  |  |  | **Max RSq** |
|  |  |  |  | 0.9777 |

**Data**

**Power Details**

Test Data

**Power**

| **α** | **σ** | **δ** | **Number** | **Power** | **AdjPower** | **LowerCL** | **UpperCL** |
| --- | --- | --- | --- | --- | --- | --- | --- |
| 0.0500 | 0.245027 | 0.021598 | 101 | 0.1418 | 0.0500 | 0.0500 | 0.8112 |

**Least Significant Number**

| **α** | **σ** | **δ** | **Number(LSN)** |
| --- | --- | --- | --- |
| 0.0500 | 0.245027 | 0.021598 | 496.8333 |

**Least Significant Value**

| **α** | **σ** | **Number** | **LSV** |
| --- | --- | --- | --- |
| 0.0500 | 0.245027 | 101 | 0.061491 |

**Response Ave PBR 5-25 Label=Sex**

**Whole Model**

| α | σ | δ | Number | Power | AdjPower | LowerCL | UpperCL |
| --- | --- | --- | --- | --- | --- | --- | --- |
| 0.05 | 0.234449 | 0.072225 | 99 | 0.7121 | 0.5142 | 0.0527 | 0.9994 |
|  |  |  |  |  |  |  |  |
| α | σ | δ | Number(LSN) | |  |  |  |
| 0.05 | 0.234449 | 0.072225 | 85.86412 |  |  |  |  |

Power details for MAP

| α | σ | δ | Number (LSN) |
| --- | --- | --- | --- |
| 0.05 | 13.52265 | 2.640009 | 103.2413 |

| α | σ | δ | Number | Power | Adj Power | Lower CL | Upper CL |
| --- | --- | --- | --- | --- | --- | --- | --- |
| 0.05 | 13.52265 | 2.640009 | 101 | 0.4933 | 0.378 | 0.05 | 0.9743 |
