# Supplementary material for: Rapid Patient-Side Evaluation of Endothelial Glycocalyx Thickness in Healthy Sedated Cats Using GlycoCheck® Software
Source: Front Vet Sci. 2022 Jan 3;8:727063. doi: 10.3389/fvets.2021.727063 (PMC8761653; doi:10.3389/fvets.2021.727063)
Supplement: Supplementary file 7 [file Data_Sheet_5.doc]

**Response Data Label=Median P50**

**Whole Model**

**REML Variance Component Estimates**

| **Random Effect** | **Var Ratio** | **Var Component** | **Std Error** | **95% Lower** | **95% Upper** | **Wald p-Value** | **Pct of Total** |
| --- | --- | --- | --- | --- | --- | --- | --- |
| ID | 0.5482353 | 0.2917241 | 0.069278 | 0.1559417 | 0.4275064 | <.0001* | 35.410 |
| Label 4[ID] |  | 0.5321147 | 0.0542929 | 0.4398913 | 0.6568865 | <.0001* | 64.590 |
| Total |  | 0.8238387 | 0.0761163 | 0.6928661 | 0.9960206 |  | 100.000 |

-2 LogLikelihood = 740.18743196

Note: Total is the sum of the positive variance components.

Total including negative estimates = 0.8238387

Residual is confounded with Label 4[ID] and has been removed.

**Response Data Label=PBR 10-19**

**Whole Model**

**REML Variance Component Estimates**

| **Random Effect** | **Var Ratio** | **Var Component** | **Std Error** | **95% Lower** | **95% Upper** | **Wald p-Value** | **Pct of Total** |
| --- | --- | --- | --- | --- | --- | --- | --- |
| ID | 0.397329 | 0.0481092 | 0.0135221 | 0.0216065 | 0.074612 | 0.0004* | 28.435 |
| Label 4[ID] |  | 0.1210816 | 0.0124261 | 0.099991 | 0.1496663 | <.0001* | 71.565 |
| Total |  | 0.1691909 | 0.015132 | 0.1430496 | 0.2032565 |  | 100.000 |

-2 LogLikelihood = 292.48248725

Note: Total is the sum of the positive variance components.

Total including negative estimates = 0.1691909

Residual is confounded with Label 4[ID] and has been removed.

**Response Data Label=PBR 20-25**

**Whole Model**

**REML Variance Component Estimates**

| **Random Effect** | **Var Ratio** | **Var Component** | **Std Error** | **95% Lower** | **95% Upper** | **Wald p-Value** | **Pct of Total** |
| --- | --- | --- | --- | --- | --- | --- | --- |
| ID | 0.075078 | 0.0515914 | 0.0506327 | -0.047647 | 0.1508297 | 0.3082 | 6.983 |
| Label 4[ID] |  | 0.6871702 | 0.0722173 | 0.5649986 | 0.8539758 | <.0001* | 93.017 |
| Total |  | 0.7387616 | 0.0620204 | 0.6308201 | 0.8771484 |  | 100.000 |

-2 LogLikelihood = 731.83042032

Note: Total is the sum of the positive variance components.

Total including negative estimates = 0.7387616

Residual is confounded with Label 4[ID] and has been removed.

**Response Data Label=PBR 5-9**

**Whole Model**

**REML Variance Component Estimates**

| **Random Effect** | **Var Ratio** | **Var Component** | **Std Error** | **95% Lower** | **95% Upper** | **Wald p-Value** | **Pct of Total** |
| --- | --- | --- | --- | --- | --- | --- | --- |
| ID | 0.4189157 | 0.0121683 | 0.0032685 | 0.0057622 | 0.0185745 | 0.0002* | 29.524 |
| Label 4[ID] |  | 0.0290472 | 0.0029607 | 0.0240174 | 0.0358501 | <.0001* | 70.476 |
| Total |  | 0.0412156 | 0.0036949 | 0.0348343 | 0.0495365 |  | 100.000 |

-2 LogLikelihood = -118.6566111

Note: Total is the sum of the positive variance components.

Total including negative estimates = 0.0412156

Residual is confounded with Label 4[ID] and has been removed.

**Response Data Label=PBR 5-25**

**Whole Model**

**REML Variance Component Estimates**

| **Random Effect** | **Var Ratio** | **Var Component** | **Std Error** | **95% Lower** | **95% Upper** | **Wald p-Value** | **Pct of Total** |
| --- | --- | --- | --- | --- | --- | --- | --- |
| ID | 0.3601122 | 0.0333543 | 0.0099677 | 0.013818 | 0.0528907 | 0.0008* | 26.477 |
| Label 4[ID] |  | 0.092622 | 0.0095368 | 0.0764427 | 0.1145725 | <.0001* | 73.523 |
| Total |  | 0.1259764 | 0.0111781 | 0.1066472 | 0.1511121 |  | 100.000 |

-2 LogLikelihood = 209.67071446

Note: Total is the sum of the positive variance components.

Total including negative estimates = 0.1259764

Residual is confounded with Label 4[ID] and has been removed.

**Response Data Label=RBC filling %**

**Whole Model**

**REML Variance Component Estimates**

| **Random Effect** | **Var Ratio** | **Var Component** | **Std Error** | **95% Lower** | **95% Upper** | **Wald p-Value** | **Pct of Total** |
| --- | --- | --- | --- | --- | --- | --- | --- |
| ID | 0.4753324 | 21.823407 | 5.5865779 | 10.873916 | 32.772899 | <.0001* | 32.219 |
| Label 4[ID] |  | 45.91189 | 4.703672 | 37.926549 | 56.728967 | <.0001* | 67.781 |
| Total |  | 67.735297 | 6.1678561 | 57.103247 | 81.657246 |  | 100.000 |

-2 LogLikelihood = 2024.434958

Note: Total is the sum of the positive variance components.

Total including negative estimates = 67.735297

Residual is confounded with Label 4[ID] and has been removed.

**Response Data Label=Valid vessel density**

**Whole Model**

**REML Variance Component Estimates**

| **Random Effect** | **Var Ratio** | **Var Component** | **Std Error** | **95% Lower** | **95% Upper** | **Wald p-Value** | **Pct of Total** |
| --- | --- | --- | --- | --- | --- | --- | --- |
| ID | 1.0052017 | 3170.1821 | 611.85919 | 1970.9601 | 4369.4041 | <.0001* | 50.130 |
| Label 4[ID] |  | 3153.7772 | 322.75363 | 2605.7628 | 3895.8805 | <.0001* | 49.870 |
| Total |  | 6323.9593 | 638.82361 | 5237.3655 | 7789.6015 |  | 100.000 |

-2 LogLikelihood = 3300.589548

Note: Total is the sum of the positive variance components.

Total including negative estimates = 6323.9593

Residual is confounded with Label 4[ID] and has been removed.
